# Cyclic Stretch Promotes Cellular Reprogramming Process through Cytoskeletal‐Nuclear Mechano‐Coupling and Epigenetic Modification

**DOI:** 10.1002/advs.202303395

**Published:** 2023-09-19

**Authors:** Sung‐Min Park, Jung‐Hwan Lee, Kwang Sung Ahn, Hye Won Shim, Ji‐Young Yoon, Jeongeun Hyun, Jun Hee Lee, Sunyoung Jang, Kyung Hyun Yoo, Yoon‐Kwan Jang, Tae‐Jin Kim, Hyun Kyu Kim, Man Ryul Lee, Jun‐Hyeog Jang, Hosup Shim, Hae‐Won Kim

**Affiliations:** ^1^ Institute of Tissue Regeneration Engineering (ITREN) Dankook University Cheonan 31116 Republic of Korea; ^2^ Department of Nanobiomedical Science and BK21 NBM Global Research Center for Regenerative Medicine Dankook University Cheonan 31116 Republic of Korea; ^3^ Mechanobiology Dental Medicine Research Center Dankook University Cheonan 31116 Republic of Korea; ^4^ Department of Biomaterials Science College of Dentistry Dankook University Cheonan 31116 Republic of Korea; ^5^ Department of Regenerative Dental Medicine College of Dentistry Dankook University Cheonan 31116 Republic of Korea; ^6^ Cell & Matter Institute Dankook University Cheonan 31116 Republic of Korea; ^7^ UCL Eastman‐Korea Dental Medicine Innovation Centre Dankook University Cheonan 31116 Republic of Korea; ^8^ Laboratory of Biomedical Genomics Department of Biological Sciences Sookmyung Women's University Seoul 04310 Republic of Korea; ^9^ Department of Integrated Biological Science Pusan National University Pusan 46241 Republic of Korea; ^10^ Department of Biological Sciences Pusan National University Pusan 46241 Republic of Korea; ^11^ Soonchunhyang Institute of Medi‐Bio Science (SIMS) Soonchunhyang University Cheonan 31151 Republic of Korea; ^12^ Department of Biochemistry Inha University School of Medicine Incheon 22212 Republic of Korea

**Keywords:** cell reprogramming, epigenetic change, induced pluripotent stem cells, mechanotransduction, physical force

## Abstract

Advancing the technologies for cellular reprogramming with high efficiency has significant impact on regenerative therapy, disease modeling, and drug discovery. Biophysical cues can tune the cell fate, yet the precise role of external physical forces during reprogramming remains elusive. Here the authors show that temporal cyclic‐stretching of fibroblasts significantly enhances the efficiency of induced pluripotent stem cell (iPSC) production. Generated iPSCs are proven to express pluripotency markers and exhibit in vivo functionality. Bulk RNA‐sequencing reveales that cyclic‐stretching enhances biological characteristics required for pluripotency acquisition, including increased cell division and mesenchymal‐epithelial transition. Of note, cyclic‐stretching activates key mechanosensitive molecules (integrins, perinuclear actins, nesprin‐2, and YAP), across the cytoskeletal‐to‐nuclear space. Furthermore, stretch‐mediated cytoskeletal‐nuclear mechano‐coupling leads to altered epigenetic modifications, mainly downregulation in H3K9 methylation, and its global gene occupancy change, as revealed by genome‐wide ChIP‐sequencing and pharmacological inhibition tests. Single cell RNA‐sequencing further identifies subcluster of mechano‐responsive iPSCs and key epigenetic modifier in stretched cells. Collectively, cyclic‐stretching activates iPSC reprogramming through mechanotransduction process and epigenetic changes accompanied by altered occupancy of mechanosensitive genes. This study highlights the strong link between external physical forces with subsequent mechanotransduction process and the epigenetic changes with expression of related genes in cellular reprogramming, holding substantial implications in the field of cell biology, tissue engineering, and regenerative medicine.

## Graphical abstract

Here, mechanotransduction mechanism underlying the enhanced induced pluripotent stem cell (iPSC) production by cyclic‐stretching is demonatrated. Cyclic‐stretching activates key mechanosensitive molecules (integrins, perinuclear actins, nesprin‐2, and YAP), across the cytoskeletal‐to‐nuclear space, leading to altered epigenetic modifications and cellular reprogramming.

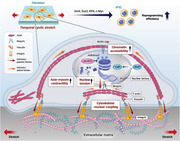

## 1 Introduction

Cells sense the physical properties of their microenvironment and translate them into biochemical signals to regulate their functions and behaviors.^[^ ^1^ ^]^ This process, called mechanotransduction, is critical in a wide range of pathophysiological conditions, including development, wound healing, and cancer progression.^[^ ^2^ ^]^ The mechanotransduction machinery encompasses the key components present in the cell membrane, within the cytosol, and even in the nucleus. These elements, including integrins, focal adhesion molecules, actomyosin, the linker of nucleoskeleton and cytoskeleton (LINC) complex, and nuclear lamina, form an interconnected network that facilitates cellular communication with the extracellular space, governing gene expressions.^[^ ^2^, ^3^ ^]^ Recent studies have elucidated the mechanisms of cellular mechanosensing through receptors as well as the intracellular mechanosignaling processes mediated by various kinases and transcription factors that are key involved in a variety of cell‐fate altering events, such as cell division, lineage specification, metastatic dissemination, and apoptotic death.^[^ ^3^, ^4^ ^]^

One of the most striking examples of cell‐fate conversion is cellular reprogramming, where cells can be transformed from one stable identity to another.^[^ ^5^ ^]^ Induced pluripotent stem cells (iPSCs) were first generated through cellular reprogramming by the forced expression of a few transcription factors, such as Oct_4_ (O), Sox_2_ (S), Klf_4_ (K) and c‐Myc (M), collectively referred to as OSKM.^[^ ^6^ ^]^ Additionally, direct reprogramming process, the conversion of one somatic cell type into another without going through iPSC, has been reported in several cell types.^[^ ^6^, ^7^ ^]^ These reprogrammed cells have immense potential for regenerative medicine and drug discovery for treating intractable diseases as they can be derived from the patient's own somatic cells. However, the generation of these cells with high efficiency remains a significant challenge in clinical translation.

Over the past decade, considerable efforts have been made to optimize the biochemical factors that regulate the transcriptional process of cellular reprogramming, including genes, proteins, and small molecules, to enhance efficiency.^[^ ^7^, ^8^ ^]^ In addition to these biochemical factors, the importance of biophysical factors, such as matrix cues and external forces, in modulating and improving the efficiency of cellular reprogramming, has also been recognized. For example, matrix‐enabled physical cues, such as parallel microgrooves and aligned polymer nanofibers, have been shown to improve the reprogramming efficiency of iPSCs derived from fibroblasts.^[^ ^9^ ^]^ Furthermore, the stiffness of substrates has been demonstrated to modulate iPSC reprogramming, with lower stiffness resulting in increased efficiency.^[^ ^10^ ^]^ Lateral confinement of cells via micropatterned substrates can also drive iPSC‐like reprogramming, even in the absence of exogenous reprogramming factors.^[^ ^11^ ^]^ Of note, such effects of matrix cues (i.e., topography, stiffness, confinement) on cellular reprogramming have been found to be mediated by epigenetic changes, including histone modifications. External forces, such as electromagnetic field and mechanical stretching, have also been shown to enhance the efficiency of cellular reprogramming, possibly through the activation of mechanotransduction signaling pathways.^[^ ^12^ ^]^ Mechanotransduction events along the cytoskeletal‐to‐nuclear axis, and the resultant epigenetic modifications, may be coordinated to contribute to the increased reprogramming efficiency, although further systematic and in‐depth investigations are required to fully understand these processes.

With these observations in mind, here we aim to enhance the reprogramming efficiency by means of introducing biophysical cues to cells, and to investigate the mechanotransduction mechanisms underlying the events. To achieve this, we employed a simple drug‐inducible OKSM transgenic system in secondary mouse embryonic fibroblasts (2^nd^ MEF),^[^ ^13^ ^]^ and applied a cyclic stretch as a physical stimulus. Our results show that temporarily‐applied cyclic stretch can enhance iPSC reprogramming efficiency up to ≈4‐fold, along with accelerated biological processes required for iPSC generation. We identified the activation of mechanosensitive molecules along the cytoskeletal‐to‐nuclear axis, such as integrins, perinuclear actin, nesprin, and yes‐associated protein (YAP). Furthermore, we found significantly altered epigenetic marks in stretched cells, primarily downregulation of H3K9 methylation and global gene occupancy change, suggesting the implication of epigenetic changes in cell‐fate conversion regulated by physical cues. These findings may facilitate the development of novel technological approaches for enhancing cellular reprogramming efficiency through biophysical modulation, as well as deepen our understanding of the mechanotransduction mechanisms involved in cellular reprogramming.

## 2 Results and Discussion

### 2.1 Cyclic Stretch Temporarily Applied During Reprogramming Process Enhances Efficiency of iPSC Generation

We utilized a previously established cell line called “2^nd^ MEF” for cellular reprogramming experiments. This cell line is a drug‐inducible transgenic system that has been engineered to express Yamanaka factors (OSKM) and can be reprogrammed to iPSCs through non‐viral drug (doxycycline) treatment.^[^ ^13^ ^]^ This system minimizes the genetic heterogeneity of cells by avoiding variations in cellular transfection ratios across experimental groups, allowing us to maximize the effects of external experimental cues (here cyclic stretch), and ensure scalability for mechanism analyses.^[^ ^21^ ^]^

**Figure 1:**
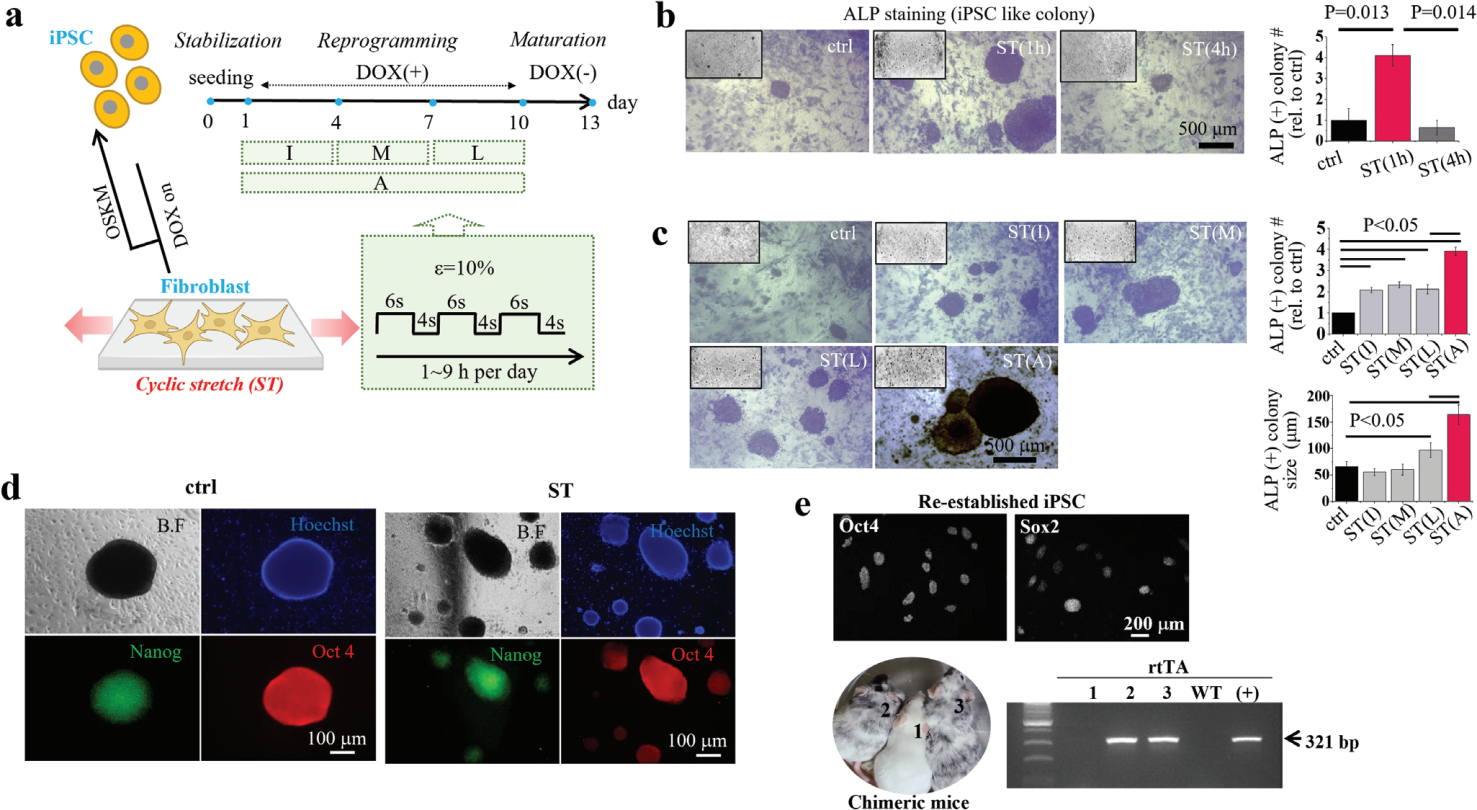
Cyclic stretch during reprogramming process enhances iPSC generation. a) Illustration showing the experimental scheme of the cyclic‐stretch‐induced cellular reprogramming. Fibroblasts (mouse embryonic fibroblasts (MEF) were used; so‐called secondary MEF, a doxycycline (DOX)‐inducible transgenic (OKSM) system), plated on a culture well for 1 day, were reprogrammed in conjunction with a cyclic stretch using a Flexell system for up to 9 days upon DOX exposure, which was followed by a maturation for 3 days without DOX. Cyclic stretch (named “ST”, with a cycle of 6‐sec/4‐sec stretch on/off) was applied for 1 to 9 h per day, and the timing of stretch was either initial 3 days (“I”), middle 3 days (“M”), last 3 days (“L”), or all 9 days (“A”). b) Representative images of ALP(+) iPSC colonies and the quantification of colony number, showing ≈4‐fold increase in ST group (1 h day^−1^ stretch for 9 days) than non‐stretched control (ctrl) group (n = 5). c) Representative images of ALP(+) iPSC colonies and the quantification of colony number and size at different stretch conditions (timing of stretch), showing the highest when stretched all 9 days (n = 5). d) Representative images of iPSC colonies immunocytochemical‐stained for iPSC markers (Nanog and Oct4). iPSC colonies in stretch condition (iPSC‐ST) co‐expressed both markers. e) Confirmation of iPSCs by various experimental approaches; Oct_4_ and Sox_2_ were expressed in re‐established iPSC from 1^st^ surrogate mice (the re‐establishment of iPSCs is illustrated in supplementary data, Figure S2, Supporting Information). Re‐established iPSCs were again injected into blastocysts and transferred to 2^nd^ surrogates to produce chimeric offspring. Coat‐color chimeras (#2 and #3) contained rtTA gene from iPSC‐ST based on PCR, indicating successful transmission of iPSC‐ST into chimeras of next generation. WT is wild type without iPSC‐ST injection and (+) is positive control for rtTA. Non‐parametric repeated measurement analysis (Friedman test) with Dunn's multiple comparison as post hoc for (B) and (C). P values (or those considered significant) are noted in the graphs.

We seeded 2^nd^ MEF on collagen (type I) coated elastic substrates and subjected them to a cyclic stretch using a Flexell system (Figure S1, Supporting Information). The cyclic stretch was designed to have a frequency of 0.1 Hz with a 6 s stretch (10% strain) followed by a 4 s rest (0% strain) in a square wave patten, and the daily duration of the cyclic stretch was varied between 1 and 9 h per day, for a maximum of 9 days, according to the schedule outlined in **Figure** **1**a. We fixed the stretch conditions at 0.1 Hz with a 40% relaxation time (4 s rest after 6 s strain) to minimize cell apoptosis while eliciting biological effects. These conditions were determined based on existing literatures and our pilot experiments.^[^ ^14^ ^]^

We first measured the efficiency of iPSC generation by counting the number of alkaline phosphatase (ALP)(+) colonies. When cells were stretched for a temporal period of 1 h per day for 9 days, the number of ALP(+) colonies increased by ≈4‐fold, however, a longer stretch of 4 h per day for 9 days did not show the same effect (Figure 1b). We further varied the duration schedule of the stretch and found that stretching overall 9 days was the most effective in terms of the number and size of ALP(+) colonies (Figure 1c).

The iPSCs obtained under the optimized stretch condition (“iPSC‐ST”) were confirmed by the co‐expression of Oct4 and endogenous Nanog (with GFP), known as a major early and late marker for iPSCs, respectively (Figure 1d). The iPSCs were further confirmed to have in vivo functional pluripotency by means of blastocyst injection and subsequent production of chimeric mice. We dissociated iPSC‐ST to single cells, injected them into blastocysts, and implanted in a uterus of surrogate mice (Figure S2a, Supporting Information). We then sacrificed the 13.5‐days‐grown embryos and reconducted reprogramming procedures using the fibroblasts derived from chimeric embryos. We confirmed a successful involvement of exogenous iPSC‐ST in embryo during development by the presence of rtTA (reverse tetracycline‐controlled transactivator), a component of a drug‐inducible transgenic system originated from iPSC‐ST, in the collected embryos by PCR (4 out of 6 embryos, Figure S3, Supporting Information). Next, we re‐established iPSCs from fibroblasts originated from the embryos with iPSC‐ST, and then confirmed the successful re‐establishment of iPSC colonies by pluripotency markers (expression of ALP, Nanog‐GFP, Oct_4_ and Sox_2_, and embryoid bodies) (Figure S4, Supporting Information). Last, we injected the re‐established iPSCs into blastocysts to produce chimeric offspring by maintaining the injected embryos to full term (Figure S2b, Supporting Information). Chimeras with coat‐color mosaicism (black coat color originated from iPSCs) were born (2 out of 3) with the presence of rtTA based on PCR, indicating successful contribution of iPSC‐ST to the next generation (Figure 1e). These results suggest that iPSC‐ST have in vivo functional pluripotency and can be used for further research.^[^ ^15^ ^]^

### 2.2 Cyclic Stretched Cells Feature Enhanced Biological Characteristics Required for iPSC Generation

**Figure 2:**
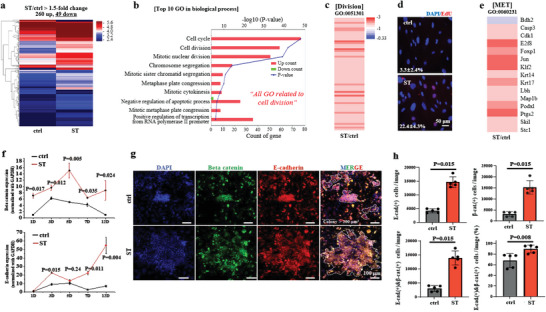
Cyclic stretch allows cells to feature enhanced biological activity for pluripotency acquisition, including increased cell division and mesenchymal‐epithelial transition (MET). a) RNA sequencing at day 2, revealing an overall transcriptome difference between groups, with 49 downregulated and 260 upregulated differentially expressed genes (DEGs) over 1.5‐fold change. b) Gene Ontology (GO) database analysis by DAVID with a total of 309 DEGs, showing the upregulated genes were mainly associated with cell cycle, cell division, mitotic nuclear division, etc. of top 10 biological processes, which highly correlated with the cell division, an essential biological activity for iPSC generation. c) Investigation of the genes related to cell division in GO, revealing that most of them were upregulated (over 1.5‐fold) by cyclic stretch. d) EdU staining of cells, confirming a higher DNA synthesis (cell division) by cyclic stretch, with an ≈7‐fold increase. e) Analysis of the genes related with MET in GO, showing that most of them were also upregulated (over 1.5‐fold) by cyclic stretch. f) Expression of epithelial markers (E‐cadherin and β‐catenin) recorded during the reprogramming process over 12 days, showing significantly higher levels in stretched cells. g) Immunostaining of cells for E‐cadherin (red) and β‐catenin (green), and DAPI‐co‐staining for nuclei at day 6, revealing a higher population of E‐cadherin(+) and β‐catenin(+) cells in stretched condition. h) Immunofluorescence‐based quantification of cells positive for E‐cadherin, β‐catenin, and both of them. Arrowed areas indicate colonies with an area exceeding 200 µm^2^, and the E‐cadherin/β‐catenin double‐positive cells were analyzed and presented in terms of both absolute numbers and fractions. Non‐parametric Mann–Whitney U test for (f) and (h). P values are noted in the graphs.

To further investigate the biological changes induced by cyclic stretch, we performed transcriptomic analyses of cells with and without stretch at day 2 using bulk RNA sequencing. We observed overall transcriptome differences between groups, including 260 upregulated and 49 downregulated differentially expressed genes (DEGs) over 1.5‐fold change (**Figure** **2**a). Gene Ontology (GO) analysis was performed on a total of 309 DEGs, which revealed that most of the top 10 biological processes, such as cell cycle, mitotic nuclear division, and mitotic cytokinesis, were highly related to cell division, an essential biological activity during iPSC reprogramming,^[^ ^16^ ^]^ as shown in Figure 2b (also in Figure S5, Supporting Information).

Top 30 GO terms in molecular function (i.e., DNA, protein, and microtubule binding) and cellular component (i.e., nucleus and chromosome) also supported the enhanced cell division activity by cyclic stretch. Kyoto Encyclopedia of Genes and Genomes (KEGG) analysis identified cell cycle and oocyte meiosis as the top two enriched up‐KEGG pathways, further reinforcing the aforementioned cellular activity. DEGs of cell division GO (GO:00 51301) showed all upregulations in stretched cells (Figure 2c), which was supported by ≈7‐fold increase (22.4% from ST versus 3.3% from control) in DNA synthesis by EdU staining (Figure 2d). These results suggest that cyclic stretch enhances cell division and may contribute to the enhanced efficiency of iPSC reprogramming. The activation of cell division by mechanical stretch has been reported elsewhere with different experimental settings,^[^ ^14^, ^17^ ^]^ and here we suggest that a similar mechano‐stimulating role of the cyclic stretch may be involved in pluripotency acquisition in fibroblasts. The possibility of spontaneous differentiation into 3 germ layers in the initial reprogramming stage under the stretching condition was ruled out, as the RNA‐seq based DEGs related with 3 germ layers were not pronounced (Figure S6, Supporting Information), which was in parallel with the previous finding.^[^ ^18^ ^]^

Next, we analyzed the mesenchymal‐to‐epithelial transition (MET) which is a key process in transformation of fibroblasts into pluripotent cells. The upregulation of DEGs in the MET GO (GO:00 60231) analysis (Figure 2e) suggests that the cyclic stretch promotes the transition of fibroblasts to epithelial‐like cells. The qRT‐PCR analysis of epithelial markers β‐catenin and E‐cadherin^[^ ^19^ ^]^ over 12 days further confirmed the upregulation of these markers in the stretched group (Figure 2f), indicating a higher degree of MET. Immunostaining of cells for both markers at day 6 also showed significantly higher expressions in terms of numbers and fractions in the stretched condition (Figure 2g,h; Figure S7, Supporting Information). These findings suggest that the activation of MET by mechanical stretch may be essentially involved in pluripotency acquisition in fibroblasts.

### 2.3 Cyclic Stretch Couples the Mechanosensitive Machineries along The Cytoskeletal‐To‐Nuclear Axis

Next, we investigated the role for mechanotransduction in enhancing the iPSC reprogramming efficiency. We hypothesized the mechanical stretch of the substrate could directly affect the adherent fibroblasts through mechanotransduction processes along the mechanosensitive machineries lying in the focal adhesion‐cytoskeleton‐nucleus axis. In fact, upon stretch, fibroblasts tended to align perpendicular to the stretch direction, implying that cells could mechanosense the physical force and then conform their shape by altering cytoskeletal organization. Of note, we found that cells could align significantly only when the stretch duration was long enough (4 and 12 h day^−1^), but not in a short duration (1 h day^−1^) (Figure S8, Supporting Information). This is intriguing because the enhanced iPSC generation was noticed only in the short duration condition of 1 h day^−1^ (as shown in Figure 1). We suggest that a narrow stretch window that is effective in driving iPSC generation through cellular mechanosensing might exist, i.e., the stretch level that can induce cellular mechanosensing temporally (and mildly) while not altering the distribution of stress fibers that are involved in cell alignment.

**Figure 3:**
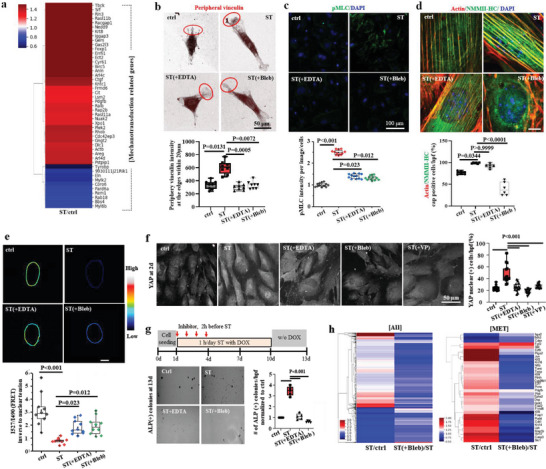
Cytoskeletal‐to‐nuclear mechanosensitive machineries involved in mechanotransduction for enhanced iPSC reprogramming. a) RNA sequencing at day 2 revealed upregulation of mechanotransduction related genes (actin cytoskeleton organisation GO:00 30036, myosin light chain kinase activity GO:0 004687, GTPase activity GO:0 003924, Rho protein signal transduction GO:0 007266, Rho‐dependent protein serine/threonine kinase activity GO:00 72518, actin‐mediated cell contraction GO:00 70252, regulation of actin polymerization or depolymerization GO:0 008064) in stretched condition (over 1.3‐fold change). b) Expression of vinculin, one of the major F‐actin associated focal adhesion complexes to transmit the external force generated by substrate stretch, at the peripheral region of long‐axis edges (within 20 µm, marked as red circles), was higher in stretched conditions, which became abrogated by the treatment of mechanotransduction inhibitors (integrin deactivator EDTA or actomyosin contractility inhibitor blebbistatin, Bleb). c) Expression of phosphorylated myosin light chain (pMLC) that controls actomyosin contractility, was upregulated in stretched cells, which became abrogated by the intervention with inhibitors. d) Expression of non‐muscle‐myosin‐heavy chain IIA (NMMHC‐IIA), one of the actin‐binding motor proteins, was enhanced in stretched condition, which decreased by the inhibitors (mainly Bleb). e) The mechanical tension surrounding the nuclear membrane was measured using a fluorescence resonance energy transfer (FRET)‐based tension biosensor employed in Nesprin‐2G, a key component of the LINC complex. Stretched cells revealed a decreased FRET score compared with control, indicating that stretch led to higher nuclear tension. f) YAP nuclear‐translocation, considered as a marker of mechanotransducer, increased in stretched cells up to ≈50%, which became abrogated with inhibitors (EDTA, blebbistatin, or VP) treatment. g) ALP(+) colonies stimulated by a cyclic stretch was diminished by inhibitors (EDTA or blebbistatin). Non‐parametric Kruskal‐Wallis test and Dunn's multiple comparison test as post hoc for (b–e); one way ANOVA and Tukey post hoc test for (f,g). h) RNA sequencing analysis revealed the intervention of mechanotransduction pathway by Bleb downregulated most of MET‐related genes (mesenchymal to epithelial transition GO:00 60231, epithelial cell maturation GO:0 002070, epithelial cell development GO:0 002064) (over 1.3‐fold change).

We then examined the DEGs in mechanotransduction‐related GO terms to identify if the temporal stretch (1 h day^−1^) could induce cellular mechanosensing. Approximately 80% genes were upregulated (over 1.3‐fold change) in stretched cells in GO terms, such as actin cytoskeleton organization, myosin light chain kinase activity, GTPase activity, Rho protein signal transduction, Rho‐dependent protein serine/threonine kinase activity, actin‐mediated cell contraction, and regulation of actin polymerization or depolymerization (**Figure** **3**a). We also observed the expression of vinculin, a major focal adhesion complex molecule that links ECM and F‐actin to transmit forces generated by substrate stretching. Stretched cells expressed higher vinculin intensity, mainly at the peripheral region (red circled) within 20 µm of the long‐axis edges (Figure 3b). Furthermore, we found that the expression of pMLC, which positively relates to actomyosin contractility to transmit mechanical forces,^[^ ^20^ ^]^ was significantly higher in stretched cells (Figure 3c). In addition, we observed that there was no significant difference in stress fibers between the stretched and control groups. However, there was a notable difference in the expression of perinuclear actin (actin cap) formation, a phenotypic F‐actin feature of cellular mechanosensing adjacent to nucleus (Figure 3d).^[^ ^21^ ^]^ Almost all cells in the stretched group (≈100%) displayed actin cap formation compared to only ≈80% in the control group. This suggests that reprogramming fibroblasts are able to mechanosense the temporal stretch cue and transmit the signal along the focal adhesion‐to‐actin axis through mechanosensitive machineries (i.e., vinculin, NMMHC‐IIA, actin cap), and then possibly into the nucleus.^[^ ^37^ ^]^ We further investigated the role of the activated mechanosensitive machineries by pharmacological inhibition using EDTA (for focal adhesion complex inhibition by extracellular calcium chelating and integrin clusters catastrophe) and blebbistatin (Bleb) (for non‐muscle myosin II inhibition). The experimental schedule for these inhibitors is shown in Figure S9 (Supporting Information), and the doses used were nontoxic to cells's total RNA transcription (Figure S10, Supporting Information). The expression of β‐tubulin molecule, which comprises another cytoskeletal component microtubule, was not significantly altered by the stretch (Figure S11, Supporting Information). This suggests that there was a more dominant change in F‐actins, mainly actins near the nucleus (vs microtubules), responding to temporal cyclic stretch during cellular reprogramming.

Next, we analyzed the mechanosensitive molecules that link cytoskeletal actins to the nucleus. We designed a FRET‐based nesprin‐2 tension sensor system to investigate the interaction between Nesprin‐2 and F‐actin, which physically links F‐actin to the nuclear envelope (schemed in Figure S12, Supporting Information).^[^ ^22^ ^]^ The FRET signal was significantly reduced in stretched cells, indicating that tight interaction and high tension had developed in the Nesprin‐2/F‐actin complex due to cyclic stretch (Figure 3e). The treatment of inhibitors (EDTA and Bleb) compromized this interaction. We also examined the mechanotransducer YAP, a transcription factor that translocates to the nucleus in response to mechanical stress.^[^ ^14^, ^23^ ^]^ Enhanced fraction of YAP‐nuclear‐located cells (up to ≈50%) was observed in stretched condition (Figure 3f), which suggests a nuclear‐cytosol coupling through a mechanosensitive transcription factor.^[^ ^24^ ^]^ The expression of YAP target genes (AREG, CTGF, and CYR61) was also upregulated in stretched cells (1.3–2.5 fold changes), as deduced from the RNA sequencing. Treatment with inhibitors, including EDTA, Bleb, and YAP deactivator, verteporfin (VP), retrieved the YAP‐nuclear‐located cells almost down to a basal level (≈20%). Interestingly, the expression of lamin A/C, a key mechanosensor in the nucleus, was not significantly altered by the stretch (Figure S13, Supporting Information). Lamin A/C is known to develop in response to mechanical stress, including stretched force,^[^ ^25^ ^]^ and is considered a key nuclear mechanosensor by adjusting nuclear tension and transmitting signals physically to chromatin,^[^ ^20^ ^]^ therefore, the expression of lamin A/C is the cellular action for nuclear resistance to deformations by stretch.^[^ ^26^ ^]^ However, it should be borne in mind that the nuclear resistance to large deformations (by high stretch) is dominated by the lamin A/C expression, whereas under stretch for small deformations (<30% of the original length of the nucleus), the nuclear resistance is dominated by chromatin organization.^[^ ^27^ ^]^ The unaltered lamin A/C expression, together with the minimal cytoskeletal reorientation (as shown in Figure S8, Supporting Information), implies that the cyclic stretch used in the study is very mild but clearly mechanosensed and transmitted to the nucleus through some key mechanosensitive machineries, including integrins, NMMHC‐IIA, actin cap, nesprin‐2, and YAP.

The pharmacological inhibition of the stretch‐sensing (substrate adhesion) and signal‐transmission (actomyosin contractility) machinery (respectively by EDTA and Bleb) significantly downregulated the stretch‐activated cellular reprogramming process, as deduced by the reduced number of ALP(+) colonies (Figure 3g). Also, the inhibition of YAP signaling (by VP treatment) was shown to downregulate the stretch‐activated cellular reprogramming (down to a level of ≈20% of stretched group, being almost close to unstretched control, as shown in Figure S14, Supporting Information), which is comparable to the effect of other mechano‐machinery inhibitors. RNA‐seq analysis further revealed profound downregulations of overall and MET‐related transcriptome profiles by the inhibition with Bleb (Figure 3h). Principal component analysis (PCA) of the 54 DEGs among three groups by RNA‐seq showed that Bleb‐treated group is relatively close to control (w/o stretch), indicating a partial recovery (≈68%) of transcriptional distance (Figure S15, Supporting Information). Further analysis of the DEGs between stretch/control and stretch(+Bleb)/stretch revealed 149 genes contra‐regulated and top 10 biological processes by GO analysis, which were identified to relate with transcriptional process and cellular contraction (Figure S16, Supporting Information). In particular, analysis of the transcriptome change (over 1.3‐fold) related with F‐actin organization showed that the widespread upregulation by the cyclic stretch was reversed by the Bleb treatment (Figure S17, Supporting Information).

### 2.4 Stretch‐Mediated Mechano‐Coupling Drives Chromatin Modification, Mainly H3K9me3 Down‐Regulation, and Its Global Gene Occupancy Change

**Figure 4:**
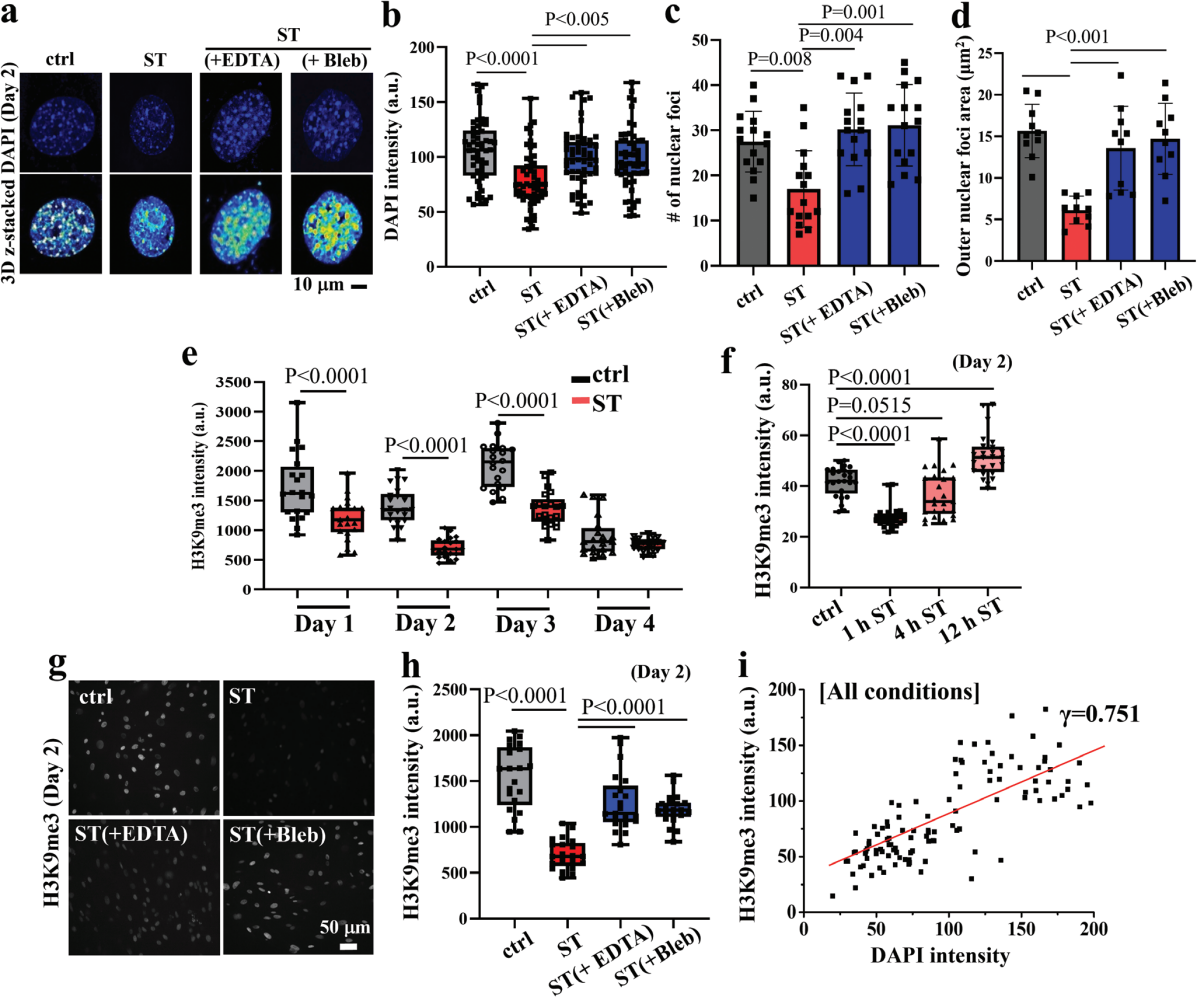
Stretch‐mediated mechanotransduction to nucleus decondenses chromatin, mainly downregulating H3K9me3. a) Representative 3D z‐stack images, visualized with maximum intensity projection (with rainbow color coded intensity), and the semi‐quantification of b) DAPI intensity, c) number of nuclear foci (with area over 1 µm^2^), and d) outer nuclear foci area anchoring nuclear lamina, as markers of heterochromatin structure. Treatment of inhibitors (EDTA and Bleb) abolished the changes in stretch‐induced heterochromatin structure. e) Effect of cyclic stretch (1 h day^−1^) on H3K9me3 expression, as analyzed daily for up to 4 days. H3K9me3 expression was significantly downregulated by stretch for up to initial 3 days, particularly at day 2. f) Effect of stretch duration (1, 4, and 12 h day^−1^) on the H3K9me3 expression. Only a temporal stretch for 1 h day^−1^ downregulated the expression. g,h) Effect of treatment of inhibitors on the H3K9me3 expression at day 2, abolishing the stretch‐induced downregulation of H3K9me3. i) A linear correlation was noted between H3K9me3 and DAPI intensity (Pearson's correlation value γ = 0.751), revealing H3K9me3 is closely related with chromatin opening by stretch‐mediated cellular reprogramming process. One‐way ANOVA and Tukey post hoc test for (b,c,d,f,h), and T‐test for (e).

Epigenetic modification is a key biological event in iPSC reprogramming process and the physical forces transmitted through actin cap can modulate such modification.^[^ ^11^, ^28^ ^]^ One of the early changes in iPSC reprogramming is the transition of the chromatin state to a globally‐open, transcriptionally‐accessible euchromatin state.^[^ ^29^ ^]^ In this study, we examined the effect of cyclic stretch on chromatin changes using nucleus DNA staining with DAPI (**Figure** **4**a). Results showed that cyclic stretch induced changes in the nucleus, including the decrease in DAPI intensity, number of nucleus foci (over 1 µm^2^), and area of nucleus foci at the periphery, all of which characterize heterochromatin structure (Figure 4b–d). These changes were independent of nucleus area and elongation factor (Figure S18, Supporting Information) and were abolished by the treatment with inhibitors (EDTA and Bleb), demonstrating the mechano‐coupling of cytoskeletal mechanosensitive machineries and nuclear component chromatin.

We also tracked the time‐dependent expression of H3K9me3 (Figure 4e) because it is a known marker of globular heterochromatin and is considered a critical epigenetic barrier against iPSC reprogramming.^[^ ^30^ ^]^ Therefore, mitigating H3K9me3 was proven to be necessary in the early stages of reprograming.^[^ ^31^ ^]^ Daily monitoring of H3K9me3 expression for 4 days (starting right after the 1 h temporal stretch) during the relatively early reprogramming stage showed significant down‐regulation for up to 3 days compared to the unstretched control, with the greatest down‐regulation observed on day 2 (Figure 4e; Figure S19, Supporting Information). By day 4, the H3K9me3 expression in the stretched group became comparable to that in the unstretched control due to the substantial decrease that occurs during this culture time point, which is attributed to the fact that iPSC reprogramming progresses with a simultaneous reduction in H3K9me3.^[^ ^32^ ^]^ Of note, the down‐regulation was only effective when the stretch was temporally applied, specifically for 1 h per day (Figure 4f; Figure S20, Supporting Information). This phenomenon was found to parallel with the change in cell alignment and ALP colony formation (aforementioned in Figure 1b; Figure S8, Supporting Information). The downregulation was then abolished when the cells were treated with mechanotransduction inhibitors (Figure 4g,h), indicating that the external stretch was directly regulating the epigenetic change in reprogramming cells. This epigenetic regulation of the external stretch has also been reported in the lineage specification of stem cells, where the stretch‐dose‐dependent H3K9 methylation varied in a biphasic manner.^[^ ^41^ ^]^ The H3K9 methylation, among other histone modifications, is a key epigenetic barrier against iPSC reprogramming,^[^ ^33^ ^]^ thus the significant decrease in H3K9me3 induced by the stretch could enable chromatin opening for cellular reprogramming. We also observed a close correlation between H3K9me3 expression and DAPI intensity (with correlation factor γ = 0.751) (Figure 4i).

### 2.5 Chromatin Immunoprecipitation Sequencing Reveals Stretch‐Mediated H3K9me3 Epigenetic Marks Implicated in Enhanced Reprogramming

**Figure 5:**
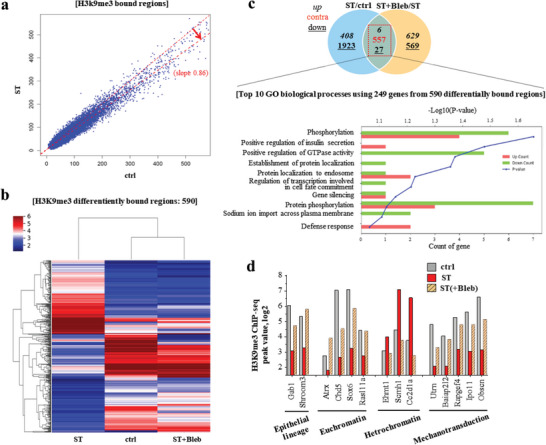
ChIP‐seq analyses of H3K9me3, revealing stretch‐mediated differentially bound regions and specific genes. a) Stretched condition (ves ctrl) showed decreased binding regions (DBRs) for widespread genes in H3K9me3. b) Heat map analysis of 590 DBRs in H3K9me3 with a fold change over 1.5, between groups (ctrl, ST, and ST(+Bleb)). Blebbistatin treatment reversed the change in DBRs, displaying close clustering map between ctrl and ST(+Bleb). c) Venn diagram of DBRs, showing 6 up‐, 27 down‐, and 557 contra‐regulated, and the top 10 biological processes identified from GO term analysis of the 290 genes from 590 DBRs. d) Stretch‐induced H3K9me3‐meidated epigenetic change was investigated at the single‐gene level, revealing some representative genes related to epithelial lineage, euchromatin/heterochromatin structure, and mechanotransduction.

We next sought to identify how the H3K9me3‐related epigenetic changes in the early stage of stretch‐induced iPSC reprogramming can affect the gene expression levels. For this, we performed genome‐wide chromatin immunoprecipitation sequencing (ChIP‐seq) analysis using an H3K9me3 antibody. DNA was pulled down using the antibody and sequenced by Illumina sequencing (NextSeq 500). The BWA algorithm was used to map the genome, and peak calling was performed by SICER 1.1.^[^ ^34^ ^]^ We used pooled input as a control, which showed no peaks in merged regions, but high non‐specific peaks in promotor and gene body regions compared to the H3K9me3‐pulled‐down groups, confirming the success of the ChIP‐seq analysis using H3K9me3 (Figure S21, Supporting Information). We detected fewer regions of H3K9me3‐enriched DNA globally in stretched cells than in non‐stretched cells (**Figure** **5**a). We also identified 590 differentially bound regions (DBRs) of H3K9me3 that showed over 1.5‐fold change in comparisons of “ST” and “Ctrl” as well as of “ST(+Bleb)” and “Ctrl” (Figure 5b). Bleb treatment retrieved the stretch‐induced H3K9me3 occupancy regions, making them more similar to the unstretched control cells, as shown by the clustering map and PCA analysis (Figure S22, Supporting Information). We next performed GO analysis using the DAVID database for the 249 genes among the 590 DBRs, which yielded the top 10 enriched terms for biological processes, molecular functions, and cellular components (Figure 5c; Figure S23, Supporting Information). Many of the top 10 terms in the biological process, molecular function, and cell component categories were related with cell signaling (phosphorylation, signal transducer activity, kinase activity), cell division (nucleotide binding, membranes), and epigenetic changes (gene silencing, demethylase activity). Our findings indicate that the epigenetically (H3K9me3‐involved) differently regulated genes by physical stretch are associated with essential biological traits of reprogramming cells, including cell signaling, cell division, and epigenetic change. Moreover, the combined analysis of bulk RNA‐seq and ChIP‐seq data revealed 29 upregulated genes with reduced H3K9me3 occupancy (Figure S24, Supporting Information). These genes were found to be associated with GO terms related to proliferation, pluripotency, and mechanotransduction. The results support that the enhanced reprogramming process induced by cyclic stretch is dependent on H3K9me3.

We further explored the stretch‐induced H3K9me3‐related epigenetic changes (> 1.5‐fold) at a single‐gene level, by analyzing the ChIP‐seq reads and peaks using UCSC genome browser. Specifically, we focused on genes related to epithelial lineage, euchromatin/heterochromatin structure, and mechanotransduction (Figure 5d). We also provided representative ChIP‐seq reads and peaks via UCSC genome browser (Figure S25, Supporting Information). We observed a stretch‐induced decrease in H3K9me3 occupancy at the promoters or bodies of genes associated with epithelial lineage (Gab1 and Shroom3), indicating acceleration of the MET process. Meanwhile, the H3K9me3 occupancy for euchromatin genes (Atrx, Chd5, Sox6, and Rasl11a) increased, along with a decrease in heterochromatin genes (Ehmt1, Scmh1, and Ash1l), indicating epigenetic changes toward open and accessible chromatin due to the stretch.^[^ ^35^ ^]^ Among these genes, Atrx is well known to be involved in transcriptional regulation and chromatin remodeling to promote euchromatin status via H3K9me3 downregulation, which supports the H3K9me3 downregulation by stretch.^[^ ^36^ ^]^ Ehmt1 and Ash1l are widely recognized histone methyltransferase, while Scmh1 is a polycomb protein predicted to have chromatin binding acitivity and negative regulation of transcription.^[^ ^37^ ^]^ Furthermore, the stretch decreased the H3K9me3 occupancy for mechanotransduction genes (Utrn, Baiap2l2, Rapgef4, Ipo11, and Obscn), where Baiap2l2 allows F actin organization, Utrn plays a role in anchoring the cytoskeleton to the plasma membrane, Obscn regulates Rho protein signal transduction, Ipo11 functions in nuclear protein import as a nuclear transport receptor, and Rapgef4 is a small GTPase, possibly mediating physical signal transduction. These results support the notion that the alteration in genes activated by cyclic stretch is closely related to H3K9me3 epigenetic modification. Furthermore, the inhibition with Bleb reversed the stretch‐induced H3K9me3 gene‐occupancy‐profile back to the unstretched control level.

Overall, the findings suggest that mechanical cues such as cyclic stretch can induce epigenetic changes that may play a crucial role in regulating gene expressions in reprogramming cells. The results of the ChIP‐seq analysis reveal that the stretch‐induced H3K9me3‐related epigenetic change is linked to alterations in the expression of genes involved in MET, chromatin opening, and mechanotransduction. This suggests that mechanical cues are capable of regulating the expression of key genes involved in cell reprogramming, and that the mechanotransduction process may be an important regulator of these events. Moreover, the findings suggest that actomyosin contractility may play a role in the physical alteration of chromatin structure induced by cyclic stretch. This observation is supported by the fact that treatment with Bleb, which disrupts actomyosin contractility, reversed the stretch‐induced effects on gene occupancy in H3K9me3. Additionally, the possible involvement of other biochemical signaling pathways, such as the YAP pathway,^[^ ^24^ ^]^ in the regulation of gene expression by mechanical cues cannot be ruled out. Overall, these findings provide new insights into the role of mechanical cues in cellular reprogramming and may have important implications for the development of new strategies for reprogrammed cell‐based therapies.

### 2.6 Single Cell RNA Sequencing Identifies Mechano‐Activated iPSC Cluster in Stretched Cells

**Figure 6:**
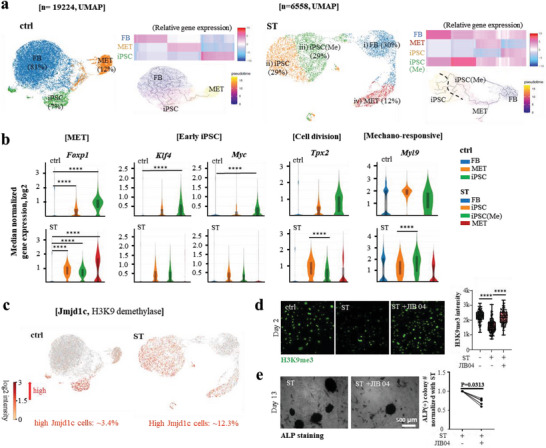
Single cell RNA sequencing to identify stretch‐activated cell clusters during reprogramming. a) Single‐cell RNA sequencing of unstretched control (ctrl) and stretch (ST) group at day 2 identified three segregated cell clusters in ctrl (FB 81%, MET 12%, and iPSC 7%) whereas four clusters in ST (FB 30%, MET 12%, iPSC 29%, and iPSC(Me) 29%) with preservation of global distances. The cell‐clusters assigned as MET highly express Foxp1 and Ctnnb1 (β‐catenin), and those as early iPSC highly express MET markers and Klf4 and Myc. Specifically, the stretched cells were identified to have two distinct sub‐clusters in early iPSC based on GO analysis by DAVID (using 50 differentially up‐regulated genes in each sub‐cluster versus others), namely “mechano‐responsive (iPSC(Me))” and “cell‐dividing (iPSC)”. 19 224 (ctrl) and 6558 (ST) single cells were analyzed^[^ ^38^ ^]^ using unsupervised clustering analysis and Uniform Manifold Approximation and Projection (UMAP) method with K‐means based clustering.^[^ ^39^ ^]^ b) Expression of representative genes related with MET (Foxp1), early iPSC (Klf4, Myc), cell division (Tpx2), and mechano‐responsive (Myl9). c) Single cell analysis of Jmjd1c (JmjC‐domain‐containing protein 1c), a well‐known H3K9 methylation eraser in iPSC reprogramming, was noted to be expressed highly in MET and cell‐dividing MET‐iPSCs clusters, with the difference as high as ≈4‐fold (≈12.3% in ST vs ≈3.4% in ctrl). d) Jmjd1c inhibitor (JIB04, 1 nM) treatment counteracted the effect of stretch in down‐regulating H3K9me3. e) ALP positive colony numbers decreased by the Jmjd1c inhibition with JIB04 treatment from six independent experiments. Data was shown after normalization for each stretching group independently. *P < 0.05, **P < 0.01, ***P < 0.001, ****P < 0.0001 adjusted by Benjamini‐Hochberg correction for multiple tests in loupe browser (ver. 5.0.1) for (b). One‐way ANOVA and Tukey post hoc test for (d, ****, P<0.0001). Non‐prapmetric Wilcoxon matched‐pairs signed rank test was performed for (e).

We next conducted single‐cell RNA sequencing (scRNA‐seq) on day 2 using the Chromium single‐cell RNA‐seq (10x Genomics) technology. We isolated and analyzed over 6000 single cells after strict quality control and normalization analysis.^[^ ^38^ ^]^ Employing unsupervised clustering analysis and the Uniform Manifold Approximation and Projection (UMAP) method with K‐means based clustering,^[^ ^39^ ^]^ we identified three segregated cell clusters in the unstretched control versus four clusters in the stretched condition (**Figure** **6**a), while preserving global distances.

To determine the cell identities in each cluster, we used previously established cell‐type or cell‐status markers. First, we assigned cell‐clusters based on the expression levels of MET (highly expressing Foxp1 and Ctnnb1 (β‐catenin)) and early pluripotency markers (highly expressing MET markers + Klf4 and Myc) (Figure 6b; Figure S26, Supporting Information). The fractions in the control group were 12% for MET and 7% for early iPSC, whereas in the stretched group, they were 12% and 58%, respectively. The ≈8 times higher fraction of early iPSCs in the stretched condition indicates enhanced iPSC reprogramming efficiency due to the stretch. Specifically, we identified two distinct sub‐clusters in early iPSC of the stretched cells based on GO analysis by DAVID, using 50 differentially up‐regulated genes in each sub‐cluster (versus others) (Figures S27 and 28, Supporting Information), namely “mechano‐responsive (iPSC(Me))” and “cell‐dividing (iPSC)”. Further, functional markers confirmed the presence of mechano‐responsive (highly expressing Acta2 (key actin protein involved in cellular contractility), Myl9 (myosin light chain protein regulating actomyosin contractility), and Actg2 (another key actin protein involved in cellular contractility) and cell‐dividing (highly expressing Cenpe, Tpx2, and Anln) iPSC (Figures S29 and 30, Supporting Information). Cell‐division related genes (Cenpe, Tpx2, Anln) were relatively higher in early iPSC, whereas mechano‐sensitive genes (Acta2, Myl9, and Actg2) were relatively upregulated in iPSC(Me).

We further sought to identify epigenetic modifiers involved in regulating H3K9 methylation, based on the single cell RNA‐seq analysis. Specifically, we analyzed the transcriptomes of histone demethylases, which are known to play a key role in epigenetic reprogramming and H3K9me3 modification. Among them, Jmjd1c (JmjC‐domain‐containing protein 1c) is a well‐known H3K9 methylation eraser in iPSC reprogramming.^[^ ^40^ ^]^ Our analysis showed that Jmjd1c was highly expressed in MET and cell‐dividing MET‐iPSC clusters under stretch conditions, with a high absolute value (Figure 6c). Although Jmjd1c was also upregulated in the MET‐iPSC cluster under control conditions, the absolute value was relatively low. The fraction of cells expressing Jmjd1c was approximately four times higher in stretched cells (≈12.3%) compared to control cells (≈3.4%). The pharmacological inhibition of Jmjd1c using JIB04 during the stretch‐involved reprogramming process reversed the H3K9me3 level up to the level seen in the unstretched control group (Figure 6d). When the Jmjd1c transcription level (high versus low) dependent expression of the genes differentially occupied by H3k9me3 (in Figure 5d) was analyzed in single cell, it revealed few genes (Atrx, Utrn, and Rapgef4) were positively regulated by Jmjd1c particularly in the control group, partially supporting the substantial role of Jmjd1c in altering genes (Figure S31, Supporting Information). Thus, it is tempting to speculate that Jmjd1c is a key H3K9 methylation modifier (downregulator) in stretch‐involved reprogramming cells. The iPSC generation, which was enhanced by stretch, was further inhibited by JIB04 treatment, based on the result of ALP(+) colony number (Figure 6e), demonstrating that Jmjd1c is one of the key epigenetic regulators for down‐regulating H3K9 methylation and stimulating iPSC generation. The reduction in ALP(+) colony number by Jmjd1c inhibition was 20–40%, which, however, appears less pronounced when compared to the inhibitory effects (up to ≈80%) of other mechano‐machineries, including YAP and actomyosin contractility (as shown in Figure 3g; Figure S32, Supporting Information). It is postulated that targeting single epigenetic regulator may not be sufficient to fully counteract the mechano‐activated effects in the reprogramming process. Based on these findings, the investigations of the interaction between mechanosensitive molecules (such as YAP) and epigenetic regulators and their combined roles in the reprogramming process remain an intriguing avenue of further research.

With regard to other possible epigenetic modifiers, we analyzed Suv39h1, as it is considered an important H3K9‐specific methyltransferase and its inhibition has been shown to enhance the iPSC reprogramming efficiency.^[^ ^41^ ^]^ Bulk RNA‐seq analysis of methyltransferases revealed a few DEGs, but SUV39H1 itself did not show significant change, and in the single‐cell RNA‐seq analysis of high‐SUV39H1‐expressing cells, the cell fractions were not high for both groups, with only a minor increase in the stretched group (≈2% in control and ≈6% in ST) (Figure S33, Supporting Information). Taken these results, we consider that SUV39H1 may not be a key player involved in the stretch‐enhanced reprogramming process, or its role may be counteracted by the actions of other histone modifiers upon external force.^[^ ^42^ ^]^ To gain a deeper understanding of the interactive roles of histone modifiers in this context, further in‐depth investigations would be of great interest.

## 3 Conclusions

**Figure 7:**
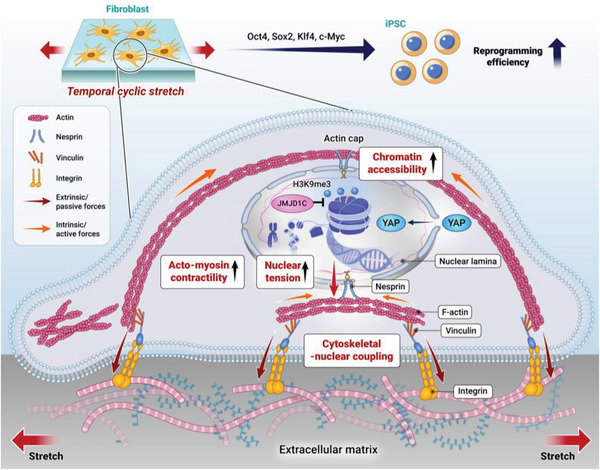
Schematic showing the mechano‐biological events during cyclic‐stretch‐mediated iPSC reprogramming process. Temporally applying cyclic stretch to fibroblasts enhances the OKSM‐induced iPSC reprogramming efficiency through the activation of mechanosensitive molecules (integrins, focal adhesions, actin cap, nesprin, and YAP) along the cytoskeletal‐nuclear axis, and the increased chromatin accessibility with downregulated H3K9me3.

Reprogramming cells with high efficiency has significant implications for regenerative therapy, disease modeling, and drug discovery. In this study, we demonstrated that the temporal application of cyclic stretch to fibroblasts during the reprogramming process substantially enhanced the efficiency of iPSC production. This enhancement was achieved through the activation of mechanosensitive molecules across the cytoskeletal‐to‐nucleus axis, including integrins, actin cap, nesprin, and YAP, as illustrated in **Figure** **7**. The stretched cells exhibited epigenetic modifications, primarily downregulation in H3K9me3, as well as global and specific changes in gene occupancy, which are implicated in the enhanced iPSC generation. Pharmacological inhibition of the mechanosensitive molecules abolished the stretching effect on the epigenetic changes and reprogramming efficiency. Through single‐cell RNA sequencing, we identified Jmjd1c as a key epigenetic modifier of stretch‐induced reprogramming cells, leading to the downregulation of H3K9me3 and thus the enhanced generation of iPSCs. Collectively, these findings highlight the strong link between the external physical forces with related mechanotransduction process and the resulting epigenetic changes with expression of related genes in cellular reprogramming. This study may provide insight into the biophysical roles underlying cellular reprogramming and optimization of mechanical cues for cell‐engineering applications in tissue regeneration and disease modeling.

## 4 Experimental Section

### iPSC Generation and Mechanical Strain

Second MEF cells containing doxycycline reactive factors (Oct4, Sox2, Klf‐4, and C‐myc) were cultured in high glucose DMEM (Welgene, Kyungbook, Republic of Korea) medium containing 10% fetal bovine serum (FBS, 35‐015‐CV, Corning Incorporated, Corning, NY, USA), 0.1 mM nonessential amino acids (MEM‐NEAA; 11 140 050, Gibco Waltham, MA, US), 1% penicillin& streptomycin (15 140 122, Gibco, Waltham, Massachusetts, USA) and 2 mM Glutamax (35050‐061, Gibco) for growth. For generating iPSCs, 2^nd^ MEF (5 × 10^4^ cells) were seeded on the collagen 1 coated elastomer (6‐well UniFlex® Culture Plates, Flexcell International, Hillsborough, NC, USA) for 24 h and iPSC induction media was freshly added iPSC induction media contained Knockout DMEM (Welgene, Gyeongsan‐si, Gyeongsangbuk‐do, Republic of Korea) with 10% Knockout serum replacement (Gibco), 5% ES‐qualified FBS (TC Biologicals, Long Beach, CA USA), 0.1 mM NEAA (Gibco), 2 mM Glutamax (Gibco), 0.055 mM β‐mercaptoethanol (Gibco), and 2 µg ml^−1^ of doxycycline (Sigma Aldrich, St. Louis, MO, USA). Cyclic uniaxial‐tension strain (square type 4s tension + 6s rest) was applied with 10% intensity, 0.1 Hz for 1, 4, or 12 h day^−1^ for the scheduled period by vacuum‐mediated tension machine (FX‐5000, Flexcell International). The strain was continuously monitored using the software provided. Doxycycline‐containing medium was replaced every 48 h until day 10. Further three days cultures were ongoing for up to 13 days without doxycycline for maturation. When inhibitors such as EDTA (1.25 mM, Sigma), blebbistatin (Bleb, 25 µM, Tocris, Bristol, UK), verteporfin (VP, 1 µM, Tocris), Y‐27632 (30 µM, Tocris), Latrunculin A (Lat A, 500 nM, Tocris), Jib04 (100 nM, Tocris) were treated, each inhibitor was incubated for pre‐treatment (1 h) and stretching (1 h) period for the initial 3 times (3 days) and refreshed to iPSC induction media. iPSC colonies were stained with alkaline phosphate solution (ALP solution, Sigma Fast BCIP/NBT, Darmstadt, Germany) for visualizing colony with iPSC like cells. The number of ALP positive colonies over 100 µm diameter was manually counted and normalized to the average number of control groups in each experiment set to display relative colony formation.

### Immunocytochemistry Analysis and Quantification

At the determined time, cells were washed with phosphate buffer saline (PBS, BPB‐9121‐004LR, T&I, Seoul, Republic of Korea) or HBSS (LB 003‐02, Welgene, with Ca^2+^ and Mg^2+^ for maintaining focal adhesion complex) and fixed with 4% paraformaldehyde solution (T&I) for 10 min at room temperature. Permeabilization was conducted with 0.2% triton X‐100 (T8787, Sigma Aldrich) for 5 min, and washed 3 times with PBS, and then immersed with 1% BSA (SM‐HEP‐250, Solmate) for 30 min for blocking procedures. All primary antibodies listed in Table S1 (Supporting Information) were diluted in 1% BSA solution and incubated overnight at 4 °C. After 3 times washing, FITC or TRITC conjugated 2^nd^ antibodies (711‐095‐152, 715‐095‐150, 711‐025‐152, 715‐025‐150, Jackson ImmunoResearch, West grove, PA, USA) were incubated for 1 h. Cell images were captured using a fluorescent microscope (Olympus, IX71, Olympus, Tokyo, Japan) or a confocal microscope (63x, Zeiss, LSM 700, Oberkochen, Germany). Cell nuclear was stained with 4′,6‐Diamidine‐2′‐phenylindole dihydrochloride (DAPI, 1:100000, D1306, Invitrogen). In the case of Z‐stack by a confocal microscope, 1 µm slice was used with the same pinhole size. Images were taken under a confocal laser microscope (Zeiss LSM 700, Germany) using the same setting format (intensity, gain, pinhole size) for equal quantification in the same batch, and then quantified mean intensity, shape, area, and other parameters with Image J (NIH, 1.52a). A number of cells for analysis was depicted in a dot. Batch variation was normalized by control cell intensity. For making a correlation graph using mean intensity between two proteins visualized by immunocytochemistry analysis, an equal number (n = 20) was randomly plotted from all groups, including the inhibitor‐treated one. Cell population quantification from the stained image was performed with the highly magnified image at 20x (40x with 0.5x digital zoom) with a Z‐stack image. All image semi‐quantification was performed after excluding dividing cells or cells with abnormal nucleus morphology. To investigate global RNA transcription level, EU (5‐Ethynyl Uridine) was stained and imaged according to the manufacturer's protocols.

### qPCR and Bulk RNA‐Sequencing

Total RNA was isolated using Ribospin (GeneAll, Seoul, Korea) at a determined time point. RNA quality was assessed by Agilent 2100 bioanalyser (Agilent Technologies, Amstelveen, The Netherlands), and RNA quantification was performed using ND‐2000 Spectrophotometer (Thermo Inc., DE, USA). A total 2 µg RNA was reverse‐transcribed to cDNA using a pre‐mixture (AccuPower RT PreMix, Bioneer, Korea) and oligo‐dT (Venlo, Netherlands, Qiagen) by a 2720 Thermal Cycler (Applied Biosystems) for qPCR. The quantitative mRNA expression level (n = 3) was measured with SYBR Green (Applied Biosystems) according to the manufacturer's instructions by real‐time PCR machine (StepOnePlus, Applied Biosystems). Primers used for amplification were as follows: B‐catenin F, 5′‐CTG ACC TGT AAA TCG TCC TTA G‐3′ and R, 5′‐ATT CCC ACC CTA CCA AGT‐3′, E‐cadherin F, 5′‐GCT GCT CCT ACT GTT TCT AC‐3′, and R, 5′‐TCT TCT CCA CCT CCT TCT T‐3′. After checking qPCR efficiency, the mRNA expression levels of each group were calculated as the relative fold change with respect to control after being normalized to GAPDH, based on the 2‐(delta) (delta) Ct value by StepOne software v2.3. The mean ± SD was shown after independent triplicate experiments. Ct value was extracted from the linear range of amplification.

For bulk RNA sequencing, a total RNA of 2 µg was obtained from cells with or without Bleb (25 µM) at day 2 after stretching. For each group, the cells were collected from three different batches, which were used for single RNA sequencing analysis. Libraries were prepared using the NEBNext Ultra II Directional RNA‐Seq Kit (NEW ENGLAND BioLabs, Inc., UK). The mRNA isolation was performed using the Poly(A) RNA Selection Kit (LEXOGEN, Inc., Austria). Following manufacture's instruction, the isolated mRNAs were used for the cDNA synthesis and shearing. Indexing was performed using the Illumina indexes 1–12. The enrichment step was carried out using of PCR. Subsequently, libraries were checked using the Agilent 2100 bioanalyser (DNA High Sensitivity Kit) to evaluate the mean fragment size. Quantification was performed using the library quantification kit using a StepOne Real‐Time PCR System (Life Technologies, Inc., USA). High‐throughput sequencing was performed as paired‐end 100 sequencing using NovaSeq 6000 (Illumina, Inc., USA). For RNA‐sequencing data analysis, raw sequencing data quality control was performed using FastQC (Simon, 2010). Adapter and low‐quality reads (<Q20) were removed using FASTX Trimmer (Hannon Lab, 2014) and BBMap (Bushnell, 2014). Then the trimmed reads were mapped to the reference genome using TopHat (Cole Trapnell et al., 2009). Gene expression levels were estimated using FPKM (Fragments Per kb per Million reads) values by Cufflinks (Roberts et al., 2011). The FPKM values were normalized using the Quantile normalization method using EdgeR within R (R Development Core Team, 2016). Data mining and graphic visualization were performed using ExDEGA (Ebiogen Inc., Seoul, Korea), ExDEGA graphicplus (v 2.0, Ebiogen Inc.) and DAVID (DAVID Bioinformatics Resources 6.8, NIAID/NIH)^[^ ^43^ ^]^ For getting GO, “https://www.ebi.ac.uk/QuickGO/” was used (European Molecular Biology Laboratory and European Bioinformatics Institute, Cambridgeshire, CB10 1SD, UK). The fold change used for each analysis was mentioned in the figure legend.

### Single Cell RNA‐Sequencing

To determine transcriptome change in single cell level, single‐cell RNA sequencing was performed with trypsinized live cells (2 × 10^5^–3 × 10^5^) at day 2 right after stretching. For the blebbistatin group, cells were pretreated for 2 h day^−1^ (1 h before stretching and another 1 h during stretching) for 2 days before collection. For each group, the cells were collected from three different batches, which were used for single RNA sequencing analysis. Unsupervised clustering analysis with the UMAP using 10x Loupe Browser (10X Genomics, Pleasanton, CA, USA) was initially conducted for clustering, cell‐type and other data mining and graphic visualization and ExDEGA and ExSCEA (Ebiogen Inc., Korea) and DAVID (v2022q2) analysis was performed for detail comparison.^[^ ^43^, ^44^ ^]^ In brief, library construction was performed using the 10X Chromium Single Cell 3′ Reagent Kits v3.1 (10X Genomics), and samples were sequenced by Illumina NovaSeq 6000 platform (Illumina, San Diego, CA, USA) according to the manufacturer protocols.^[^ ^45^ ^]^ The 10x Genomics standard seq protocol was followed by trimming the barcode and unique molecular identifier (UMI) end to 26 bp, and the mRNA end to 98 bp. Preliminary sequencing results were converted to FASTQ files with 10x Cell Ranger (10X Genomics, CA) and the FASTQ files were aligned to the mouse reference genome (mm10). The WinSeurat v2.1 (Ebiogen Inc., Korea) based on Seurat version 3 for QC, analysis, and trajectory of single‐cell RNA‐seq data were also used. Each condition was controlled over 80% of cell viability per each experiment by live cell counting analysis using trypan blue staining before short DNA barcode “tags” to each cell.

### Image Acquisition of the FRET Nesprin‐2G‐Tension Sensor

pcDNA Nesprin‐TS with TFP, elastin linker, and Venus were designed following the same sequence as Addgene plasmid no. 68 127 (a gift from Daniel Conway), and sequence was verified by Sanger sequencing (Bioneer, South Korea). Plasmid amplification was performed according to an established protocol.^[^ ^46^ ^]^ Briefly, bacteria were streaked onto an LB plate with the appropriate antibiotic. DNA plasmids were amplified and isolated using LB medium containing cultured bacteria with Plasmid Midi‐Prep (Qiagen, 12 145). Cells were cultured on a 6‐well tissue culture plate using the growth medium. When the confluence was accomplished up to 70%, cells were transfected with the Nesprin‐TS using Xfect Transfection Reagent (Takara, Kusatsu, Shiga, Japan) as per the manufacturer's instructions. 2–3 days later, cells were reseeded on flexcell plate and cultured overnight. After stretching with or without an inhibitor, cells were fixed during stretching and images were captured using Nikon (A1R) microscope with an x60 objective. The images were captured with lasers to excite 458 nm and emission from mTFP (494 nm) and yellow (527 nm) was obtained simultaneously. Cells that expressed the Nesprin‐TS at a high enough level to be readily visualized were selected for imaging; however, cells whose expression levels were too high or too low were avoided^4^. In addition, cells expressing the sensor in a discernible nuclear ring were imaged after excluding the cells expressing from the entire nucleus. Cells on cover glass treated with or without blebbistatin (25 µM) were used as controls to confirm the sensor working ability of FRET. FRET analysis was performed using the FRET analyzer plug‐in installed in Fiji image J (2.3.0/1.53q) by modified methodology.^[^ ^47^ ^]^

### Chromatin Immunoprecipitation

Cells were prepared according to the manufacturer's protocol using ChIP‐IT High Sensitivity® Kit (Active motif, Carlsbad, CA, USA) for ChIP‐seq analysis. Two groups following the stretch (with or without Bleb treatment) were used. For each group, the cells were collected from three different batches, which were used for single ChIP sequencing analysis. The collected cells were submersed in a growth medium containing 1% formaldehyde and incubated at room temperature for 15 min. Fixation was stopped by the addition of 0.125 M glycine. The cells were then treated with a Tissue Tearor (BioSpec Products, Bartlesville, OK, USA) and finally spun down and washed two times in PBS. Chromatin was isolated by the addition of lysis buffer provided, followed by disruption with a Dounce homogeniser. Lysates were sonicated and the DNA sheared to an average length of 300–500 bp. Genomic DNA (Input) was prepared by treating aliquots of chromatin with RNase, proteinase K and heat for de‐crosslinking, followed by ethanol precipitation. Pellets were resuspended and the resulting DNA was quantified on a NanoDrop spectrophotometer. Extrapolation to the original chromatin volume allowed quantitation of the total chromatin yield. An aliquot of chromatin 15 µg was precleared with protein A agarose beads (Invitrogen). Genomic DNA regions of interest were isolated using 10 µL of H3K9me3 antibody (39 161, Active Motif). Complexes were washed, eluted from the beads with SDS buffer, and subjected to RNase and proteinase K treatment. Crosslinks were reversed by incubation overnight at 65 °C, and ChIP DNA was purified by phenol‐chloroform extraction and ethanol precipitation. Illumina sequencing libraries were prepared from the ChIP and Input DNAs by the standard consecutive enzymatic steps of end‐polishing, dA‐addition, and adaptor ligation. After a final PCR amplification step, the resulting DNA libraries were quantified and sequenced on Illumina's NextSeq 500 (75 nt reads, single end). Reads were aligned to the mouse genome (mm10) using the BWA algorithm (default settings). Duplicate reads were removed and only uniquely mapped reads (mapping quality > = 25) were used for further analysis. Alignments were extended in silico at their 3′‐ends to a length of 200 bp, the average genomic fragment length in the size‐selected library, and assigned to 32‐nt bins along the genome. The resulting histograms (genomic “signal maps”) were stored in bigWig files. Peak locations were determined using the MACS algorithm (v2.1.0) with a cutoff of p‐value = 1e‐7. Peaks that were on the ENCODE blacklist of known false ChIP‐Seq peaks were removed. Signal maps and peak locations were used as input data to Active Motifs proprietary analysis program, which creates Excel tables containing detailed information on sample comparison, peak metrics, peak locations and gene annotations. USCS broswer (GRCm38/mm10) was used to visualize ChIP‐seq reads and peaks.^[^ ^48^ ^]^

### iPSC Isolation and Chimera Formation

Approximately 1 week after adding doxycycline, iPSC colonies were isolated and expanded in the absence of dox. The Nanog‐GFP^+^iPSCs were picked and injected into ICR blastocysts. Blastocysts were placed in a drop of M16 under mineral oil. A microinjection pipette with an internal diameter of 15–20 mm was used for iPSC injection using a micromanipulator. About Fifteen iPSCs were injected into the blastocoel, and the injected blastocysts were placed in KSOM (Specialty Media) and incubated at 37 °C until they were transferred to recipient females. Fifteen blastocysts that had been injected were transferred to the uterine horns of pseudopregnant females at 2.5 days post coitum, using a non‐surgical embryo transfer (NSET) device (Paratech, 60 010). The Animal Care and Use Committee at Dankook University, Republic of Korea approved all animal experiments (Approval no: DKU‐20‐039).

### Detection of a Drug‐Inducible Transgenic System by PCR

The drug‐inducible transgenic system was confirmed by the amplification of the rtTA gene from genomic DNA samples of establishment MEF (2^nd^ MEF), blastocyst‐injected embryos(13.5 fetuses), and tail biopsies from chimera. Genomic DNA was prepared using the DNeasy Tissue Kit (Qiagen) according to the manufacturer's protocol. For all samples, PCR was performed using Maxime™ PCR PreMix, i‐Taq (iNtRON Biotechnology) with forward 5′‐TCAATGGAGTCGGTATCGAAGG‐3′ and reverse 5′‐CTTGCTGACACAGGAACGCGAG‐3′primers that give rise to a 321 bp fragment. Thirty cycles of PCR amplification were performed as follows: denaturation at 95 °C for 20 s, annealing at 66 °C for 15 s, and extension at 72 °C for 30 s.

### Statistics

Data were shown as the mean ± standard deviation, unless otherwise indicated. GraphPad Prism 8 or Origin 8.5 was used for all statistical analyses and figure creation. Significance was claimed at *P<0.05, **P<0.01, ***P<0.001, and ****P<0.0001. For parametric analysis, student t‐test and one or two‐way ANOVA with Tukey's tests were utilized respectively for two or more than three groups comparisons. For non‐parametric analysis, Mann–Whitney U test and Kruskal‐Walis test with Dunn's multiple comparison tests as post hoc were used respectively for comparing two or more than three groups. For non‐parametric repeated measurement analysis, the Friedman test with Dunn's multiple comparisons was chosen.

## Conflict of Interest

The authors declare no conflict of interest.

## Author Contributions

S.‐M.P., J.‐H.L., and K.S.A. contributed equally to this work as co‐first authors. S.‐M.P., J.‐H.L., and H.‐W.K. conceptualized the study and designed the experiments; H.‐W.K. supervised the study; J.H., H.W.S., J.‐Y.Y., J.H.L., K.H.Y., Y.‐K.J., T.‐J.K., H.K.K., M.R.L., J.H.J., and J.‐H.L. performed the in vitro cell studies; K.S.A., and H.S. performed the animal surgery and in vivo experiments; J.H., H.W.S., J.‐Y.Y., J.H.L., K.H.Y., Y.‐K.J., T.‐J.K., H.K.K., M.R.L., J.H.J., and J.‐H.L. characterized the samples and analyzed the data; S.‐M.P. J.‐H.L., and H.S. drafted the manuscript; J.‐H.L., and H.‐W.K. wrote and edited the manuscript.

## Supporting information

Supporting Information

## Data Availability

The data that support the findings of this study are available from the corresponding author upon reasonable request.

## References

[1] S. Seetharaman , B. Vianay , V. Roca , A. J. Farrugia , C. De Pascalis , B. Boëda , F. Dingli , D. Loew , S. Vassilopoulos , A. Bershadsky , M. Théry , S. Etienne‐Manneville , Nat. Mater. 2021, 21, 366. 34663953 10.1038/s41563-021-01108-x

[2] a) M. A. Wozniak , C. S. Chen , Nat. Rev. Mol. Cell Biol. 2009, 10, 34; 19197330 10.1038/nrm2592 PMC2952188

[3] J.‐H. Lee , D.‐H. Kim , H.‐H. Lee , H.‐W. Kim , Biomaterials 2019, 197, 60. 30641265 10.1016/j.biomaterials.2019.01.010

[4] a) T. J. Kirby , J. Lammerding , Nat. Cell Biol. 2018, 20, 373; 29467443 10.1038/s41556-018-0038-y PMC6440800

[5] A. B. C. Cherry , G. Q. Daley , Cell 2012, 148, 1110. 22424223 10.1016/j.cell.2012.02.031 PMC3354575

[6] K. Takahashi , S. Yamanaka , Cell 2006, 126, 663. 16904174 10.1016/j.cell.2006.07.024

[7] a) J. Yu , M. A. Vodyanik , K. Smuga‐Otto , J. Antosiewicz‐Bourget , J. L. Frane , S. Tian , J. Nie , G. A. Jonsdottir , V. Ruotti , R. Stewart , Science 2007, 318, 1917; 18029452 10.1126/science.1151526

[8] a) R. A. Perez , S.‐J. Choi , C.‐M. Han , J.‐J. Kim , H. Shim , K. W. Leong , H.‐W. Kim , Prog. Mater. Sci. 2016, 82, 234;

[9] T. L. Downing , J. Soto , C. Morez , T. Houssin , A. Fritz , F. Yuan , J. Chu , S. Patel , D. V. Schaffer , S. Li , Nat. Mater. 2013, 12, 1154. 24141451 10.1038/nmat3777 PMC9675045

[10] B. Choi , K.‐S. Park , J.‐H. Kim , K.‐W. Ko , J.‐S. Kim , D. K. Han , S.‐H. Lee , Macromol. Biosci. 2016, 16, 199. 26439948 10.1002/mabi.201500273

[11] B. Roy , S. Venkatachalapathy , P. Ratna , Y. Wang , D. S. Jokhun , M. Nagarajan , G. V. Shivashankar , Proc. Natl. Acad. Sci. USA 2018, 115, E4741. 29735717 10.1073/pnas.1714770115 PMC6003522

[12] a) S. Baek , X. Quan , S. Kim , C. Lengner , J.‐K. Park , J. Kim , ACS Nano 2014, 8, 10125; 25248035 10.1021/nn502923s

[13] M. Wernig , C. J. Lengner , J. Hanna , M. A. Lodato , E. Steine , R. Foreman , J. Staerk , S. Markoulaki , R. Jaenisch , Nat. Biotechnol. 2008, 26, 916. 18594521 10.1038/nbt1483 PMC2654269

[14] a) Y. Cui , F. M. Hameed , B. Yang , K. Lee , C. Q. Pan , S. Park , M. Sheetz , Nat. Commun. 2015, 6, 6333; 25704457 10.1038/ncomms7333 PMC4346610

[15] a) A. Meissner , M. Wernig , R. Jaenisch , Nat. Biotechnol. 2007, 25, 1177; 17724450 10.1038/nbt1335

[16] a) S. Ruiz , A. D. Panopoulos , A. Herrerías , K.‐D. Bissig , M. Lutz , W. T. Berggren , I. M. Verma , J. C. Izpisua Belmonte , Curr. Biol. 2011, 21, 45; 21167714 10.1016/j.cub.2010.11.049 PMC3034649

[17] a) S. A. Gudipaty , J. Lindblom , P. D. Loftus , M. J. Redd , K. Edes , C. F. Davey , V. Krishnegowda , J. Rosenblatt , Nature 2017, 543, 118; 28199303 10.1038/nature21407 PMC5334365

[18] a) L. V. Greder , S. Gupta , S. Li , M. J. Abedin , A. Sajini , Y. Segal , J. M. W. Slack , J. R. Dutton , Stem Cells 2012, 30, 2596; 22948941 10.1002/stem.1216 PMC3626284

[19] S. Cao , S. Yu , Y. Chen , X. Wang , C. Zhou , Y. Liu , J. Kuang , H. Liu , D. Li , J. Ye , J. Biol. Chem. 2017, 292, 19122. 28935668 10.1074/jbc.M117.812537 PMC5704493

[20] J.‐K. Kim , A. Louhghalam , G. Lee , B. W. Schafer , D. Wirtz , D.‐H. Kim , Nat. Commun. 2017, 8, 2123. 29242553 10.1038/s41467-017-02217-5 PMC5730574

[21] S. B. Khatau , C. M. Hale , P. J. Stewart‐Hutchinson , M. S. Patel , C. L. Stewart , P. C. Searson , D. Hodzic , D. Wirtz , Proc. Natl. Acad. Sci. USA 2009, 106, 19017. 19850871 10.1073/pnas.0908686106 PMC2776434

[22] C. J. Walker , C. Crocini , D. Ramirez , A. R. Killaars , J. C. Grim , B. A. Aguado , K. Clark , M. A. Allen , R. D. Dowell , L. A. Leinwand , K. S. Anseth , Nat. Biomed. Eng. 2021, 5, 1485. 33875841 10.1038/s41551-021-00709-w PMC9102466

[23] M. Aragona , T. Panciera , A. Manfrin , S. Giulitti , F. Michielin , N. Elvassore , S. Dupont , S. Piccolo , Cell 2013, 154, 1047,. 23954413 10.1016/j.cell.2013.07.042

[24] A. A. Hartman , S. M. Scalf , J. Zhang , X. Hu , X. Chen , A. E. Eastman , C. Yang , S. Guo , Stem Cell Rep. 2020, 14, 730,. 10.1016/j.stemcr.2020.03.006 PMC7160372 32243844

[25] J. Swift , I. L. Ivanovska , A. Buxboim , T. Harada , P. D. P. Dingal , J. Pinter , J. D. Pajerowski , K. R. Spinler , J.‐W. Shin , M. Tewari , Science 2013, 341, 1240104. 23990565 10.1126/science.1240104 PMC3976548

[26] M. M. Nava , Y. A. Miroshnikova , L. C. Biggs , D. B. Whitefield , F. Metge , J. Boucas , H. Vihinen , E. Jokitalo , X. Li , J. M. García Arcos , B. Hoffmann , R. Merkel , C. M. Niessen , K. N. Dahl , S. A. Wickström , Cell 2020, 181, 800. 32302590 10.1016/j.cell.2020.03.052 PMC7237863

[27] a) Y. Kalukula , A. D. Stephens , J. Lammerding , S. Gabriele , Nat. Rev. Mol. Cell Biol. 2022, 23, 583; 35513718 10.1038/s41580-022-00480-z PMC9902167

[28] a) Q. Li , A. Kumar , E. Makhija , G. V. Shivashankar , Biomaterials 2014, 35, 961,; 24183171 10.1016/j.biomaterials.2013.10.037

[29] A. Gaspar‐Maia , A. Alajem , E. Meshorer , M. Ramalho‐Santos , Nat. Rev. Mol. Cell Biol. 2011, 12, 36. 21179060 10.1038/nrm3036 PMC3891572

[30] a) E. Apostolou , K. Hochedlinger , Nature 2013, 502, 462; 24153299 10.1038/nature12749 PMC4216318

[31] C. Wang , X. Liu , Y. Gao , L. Yang , C. Li , W. Liu , C. Chen , X. Kou , Y. Zhao , J. Chen , Y. Wang , R. Le , H. Wang , T. Duan , Y. Zhang , S. Gao , Nat. Cell Biol. 2018, 20, 620. 29686265 10.1038/s41556-018-0093-4

[32] a) A. Chitrakar , M. Noon , A. Z. Xiao , Cell Stem Cell 2022, 29, 1009; 35803220 10.1016/j.stem.2022.06.010 PMC9484580

[33] a) R. Xu , S. Li , Q. Wu , C. Li , M. Jiang , L. Guo , M. Chen , L. Yang , X. Dong , H. Wang , Cell Stem Cell 2022, 29, 1051; 35803226 10.1016/j.stem.2022.06.001

[34] C. Zang , D. E. Schones , C. Zeng , K. Cui , K. Zhao , W. Peng , Bioinformatics 2009, 25, 1952. 19505939 10.1093/bioinformatics/btp340 PMC2732366

[35] B. Papp , K. Plath , Cell 2013, 152, 1324. 23498940 10.1016/j.cell.2013.02.043 PMC3602907

[36] a) L. H. Wong , J. D. McGhie , M. Sim , M. A. Anderson , S. Ahn , R. D. Hannan , A. J. George , K. A. Morgan , J. R. Mann , K. H. Choo , Genome Res. 2010, 20, 351; 20110566 10.1101/gr.101477.109 PMC2840985

[37] a) D. Husmann , O. Gozani , Nat. Struct. Mol. Biol. 2019, 26, 880; 31582846 10.1038/s41594-019-0298-7 PMC6951022

[38] J.‐Y. Yoon , N. Mandakhbayar , J. Hyun , D. S. Yoon , K. D. Patel , K. Kang , H.‐S. Shim , H.‐H. Lee , J.‐H. Lee , K. W. Leong , H.‐W. Kim , Biomaterials 2022, 289, 121792. 36116170 10.1016/j.biomaterials.2022.121792

[39] E. Becht , L. McInnes , J. Healy , C.‐A. Dutertre , I. W. H. Kwok , L. G. Ng , F. Ginhoux , E. W. Newell , Nat. Biotechnol. 2019, 37, 38. 10.1038/nbt.4314 30531897

[40] F. Xiao , B. Liao , J. Hu , S. Li , H. Zhao , M. Sun , J. Gu , Y. Jin , Stem Cell Rep. 2017, 9, 927. 10.1016/j.stemcr.2017.07.013 PMC5599225 28826851

[41] a) T. T. Onder , N. Kara , A. Cherry , A. U. Sinha , N. Zhu , K. M. Bernt , P. Cahan , B. O. Mancarci , J. Unternaehrer , P. B. Gupta , E. S. Lander , S. A. Armstrong , G. Q. Daley , Nature 2012, 483, 598; 22388813 10.1038/nature10953 PMC3501145

[42] a) W. Liu , A. Padhi , X. Zhang , J. Narendran , M. A. Anastasio , A. S. Nain , J. Irudayaraj , ACS Nano 2022, 16, 10754; 35803582 10.1021/acsnano.2c02660 PMC9332347

[43] B. T. Sherman , M. Hao , J. Qiu , X. Jiao , M. W. Baseler , H. C. Lane , T. Imamichi , W. Chang , Nucleic Acids Res. 2022, 50, W216. 35325185 10.1093/nar/gkac194 PMC9252805

[44] a) D. W. Huang , B. T. Sherman , R. A. Lempicki , Nat. Protoc. 2009, 4, 44; 19131956 10.1038/nprot.2008.211

[45] a) A. Butler , P. Hoffman , P. Smibert , E. Papalexi , R. Satija , Nat. Biotechnol. 2018, 36, 411; 29608179 10.1038/nbt.4096 PMC6700744

[46] W. Kang , D.‐S. Lee , J.‐H. Jang , PLoS One 2015, 10, e0123402. 25901352 10.1371/journal.pone.0123402 PMC4406711

[47] a) P. T. Arsenovic , I. Ramachandran , K. Bathula , R. Zhu , J. D. Narang , N. A. Noll , C. A. Lemmon , G. G. Gundersen , D. E. Conway , Biophys. J. 2016, 110, 34; 26745407 10.1016/j.bpj.2015.11.014 PMC4805861

[48] L. R. Nassar , G. P. Barber , A. Benet‐Pagès , J. Casper , H. Clawson , M. Diekhans , C. Fischer , J. N. Gonzalez , A. S. Hinrichs , B. T. Lee , C. M. Lee , P. Muthuraman , B. Nguy , T. Pereira , P. Nejad , G. Perez , B. J. Raney , D. Schmelter , M. L. Speir , B. D. Wick , A. S. Zweig , D. Haussler , R. M. Kuhn , M. Haeussler , W. J. Kent , Nucleic Acids Res. 2022, 51, D1188. 10.1093/nar/gkac1072 PMC9825520 36420891

