# Supplementary material for: Cyclic Stretch Promotes Cellular Reprogramming Process through Cytoskeletal‐Nuclear Mechano‐Coupling and Epigenetic Modification
Source: Adv Sci (Weinh). 2023 Sep 19;10(32):2303395. doi: 10.1002/advs.202303395 (PMC10646259; doi:10.1002/advs.202303395)
Supplement: Supplementary file 1 — Supporting Information [file ADVS-10-2303395-s001.pdf]

ADVANCED SCIENCE
Open Access

Supporting Information

for *Adv. Sci.*, DOI 10.1002/advs.202303395

Cyclic Stretch Promotes Cellular Reprogramming Process through Cytoskeletal-Nuclear Mechano-Coupling and Epigenetic Modification

Sung-Min Park, Jung-Hwan Lee, Kwang Sung Ahn, Hye Won Shim, Ji-Young Yoon, Jeongeun Hyun, Jun Hee Lee, Sunyoung Jang, Kyung Hyun Yoo, Yoon-Kwan Jang, Tae-Jin Kim, Hyun Kyu Kim, Man Ryul Lee, Jun-Hyeog Jang, Hosup Shim* and Hae-Won Kim**

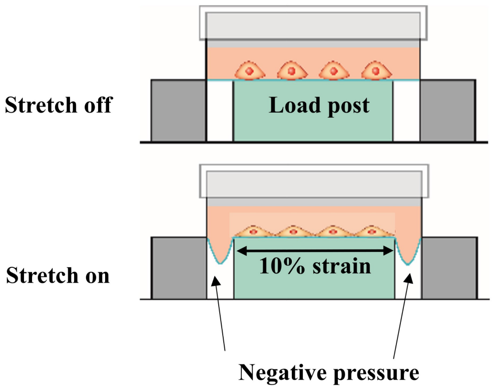

Supplementary Fig. 1. Schematic showing the cyclic stress device, Flexell system.

a

Re-establishment of reprogrammed iPSC from surrogate mouse (2nd iPSC)

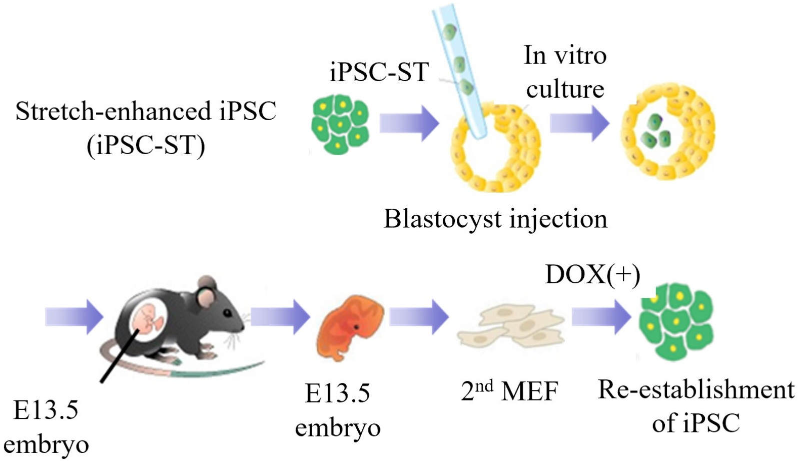

b

Newborn chimera experiment with 2nd iPSC

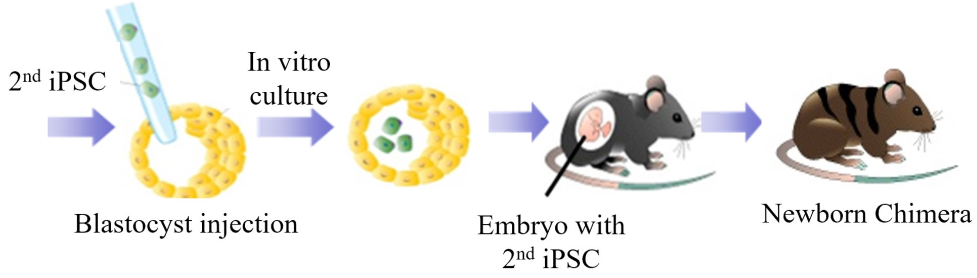

Supplementary Fig. 2. Schematic showing procedures of in vivo experiments using 'iPSC-ST'. (**a**) The re-establishment of stretch-enhanced iPSC (iPSC-ST) from surrogate mice. (**b**) The production of chimeric offspring by injection of the re-established iPSC (2nd iPSC) into blastocysts and transferring to surrogates.

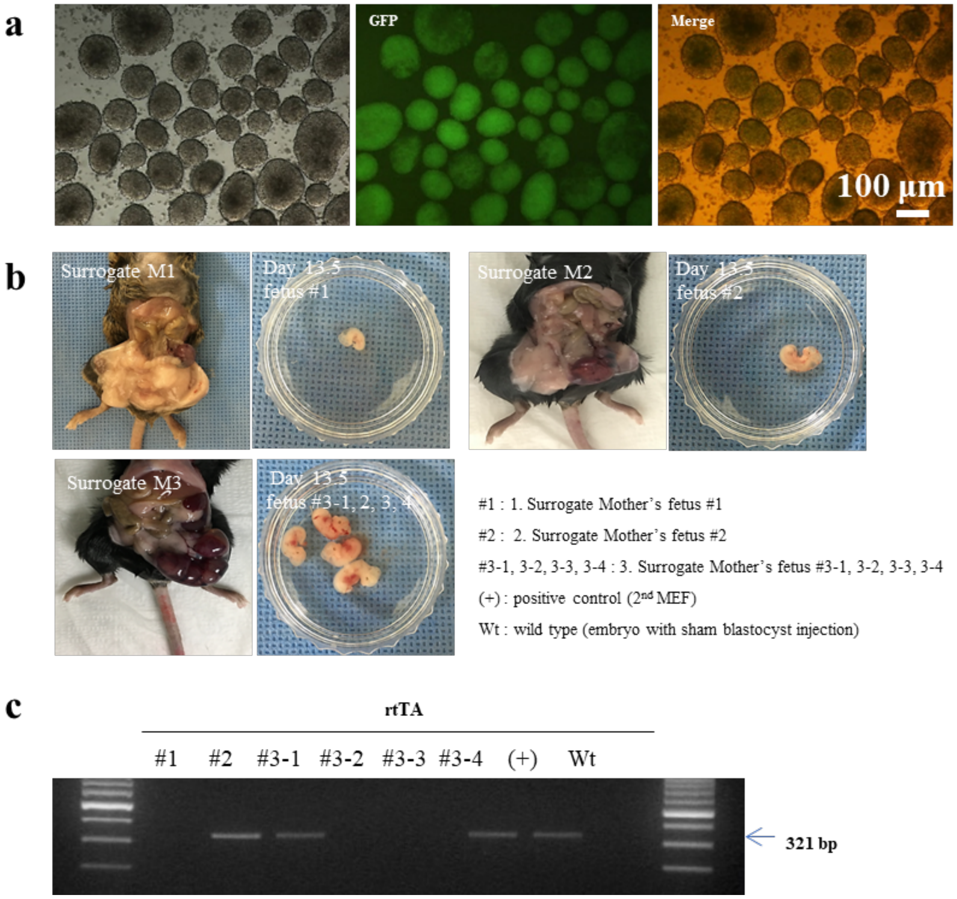

Supplementary Fig. 3. Establishment of embryonic body using stretch-enhanced iPSC (iPSC-ST) for blastocyst injection and successful reproduction of embryo. (**a**) Embryonic body of iPSC-ST cell line. Endogenous Nanog-GFP was detected from all embryonic bodies. (**b**) Collected embryo from three surrogate mothers (Surrogate M1, M2 and M3). (**c**) Detection of rtTA, a marker of exogenous iPSC-ST, in collected embryo.

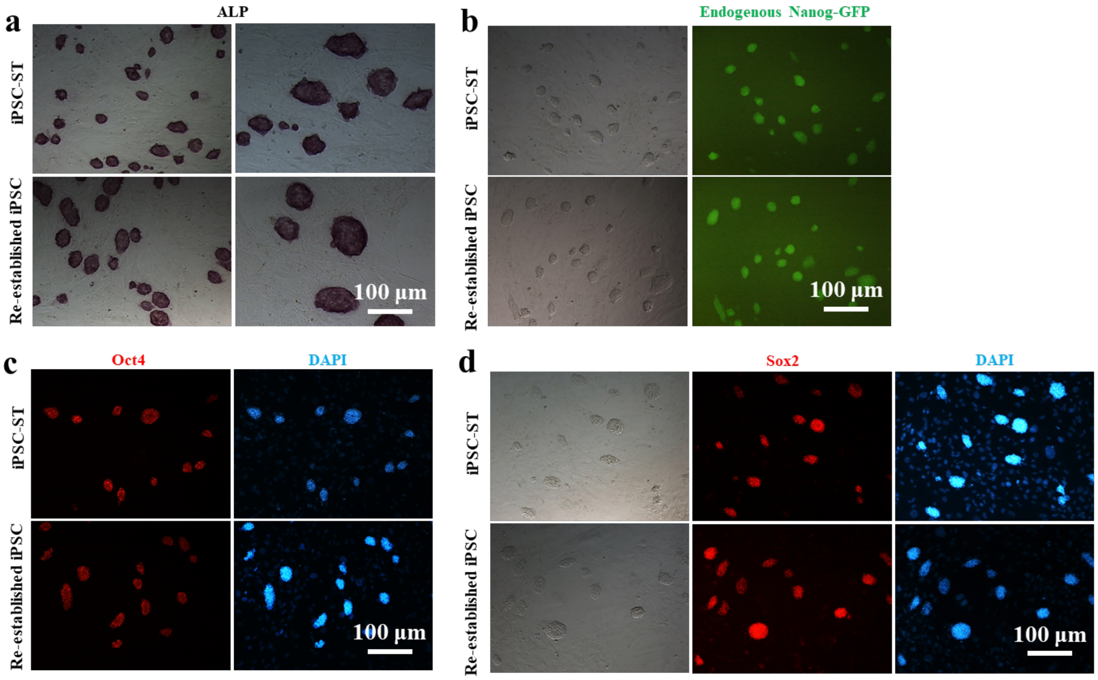

Supplementary Fig. 4. Confirmation of iPSC markers using the re-produced cells. iPSCs re-established from the iPSC-ST were successfully confirmed by the expression of pluripotent markers of colonies (**a**. ALP positive embryonic body, **b**. Nanog-GFP, **c**. Oct4, and **d**. Sox2).

Biological process

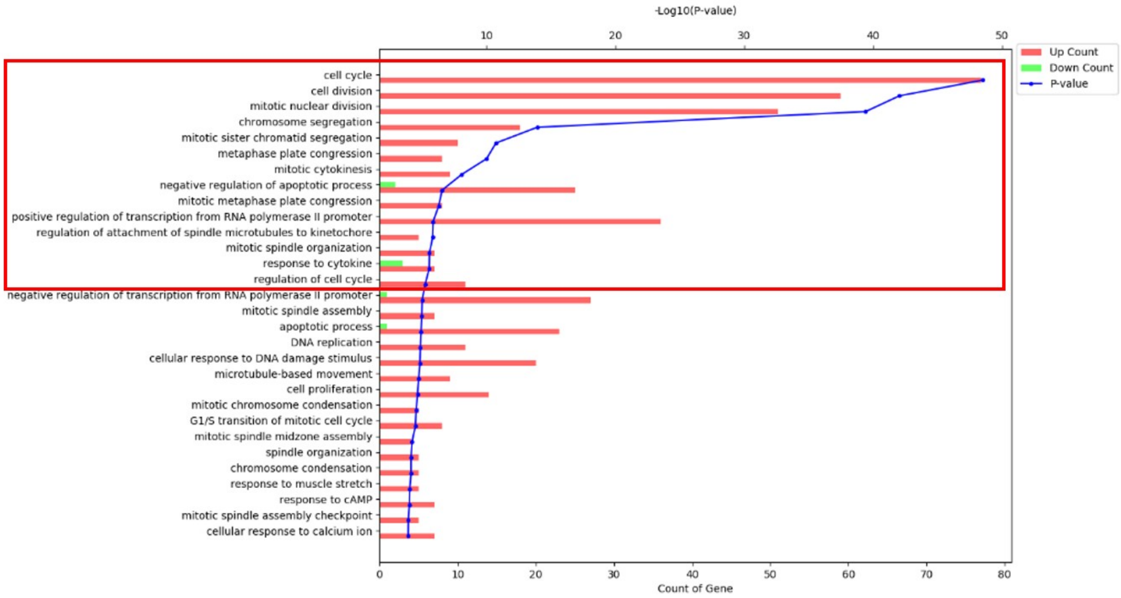

Molecular function

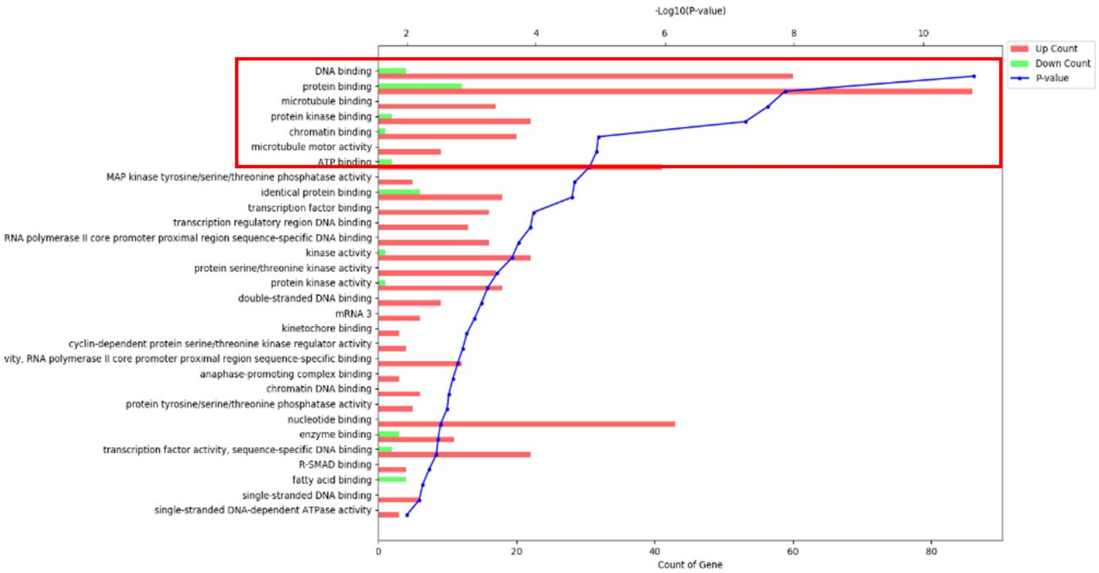

Supplementary Fig. 5. Continued.

Cellular component

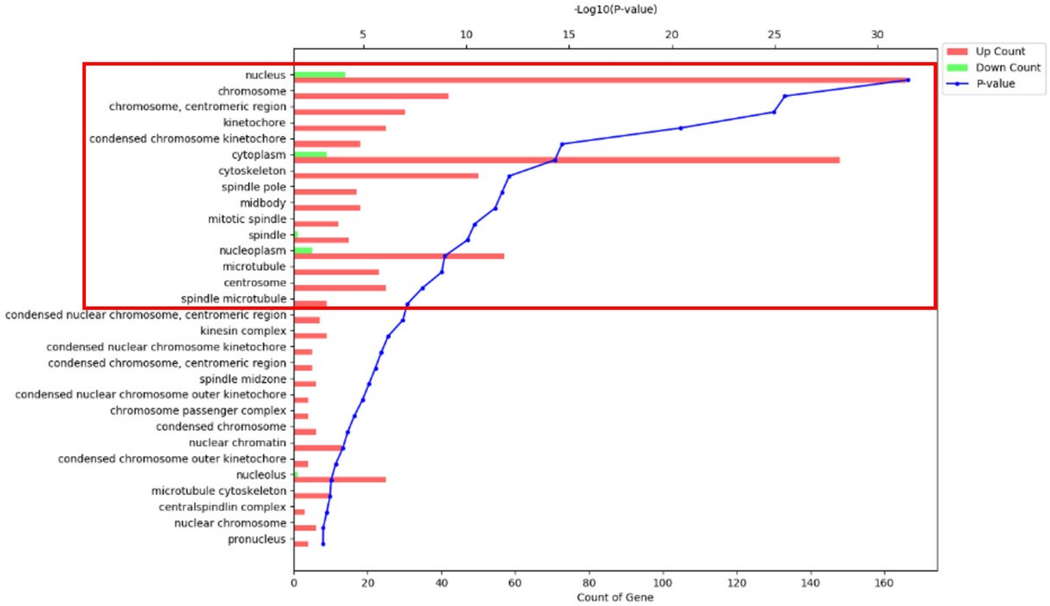

KEGG (Kyoto Encyclopedia of Genes and Genomes)

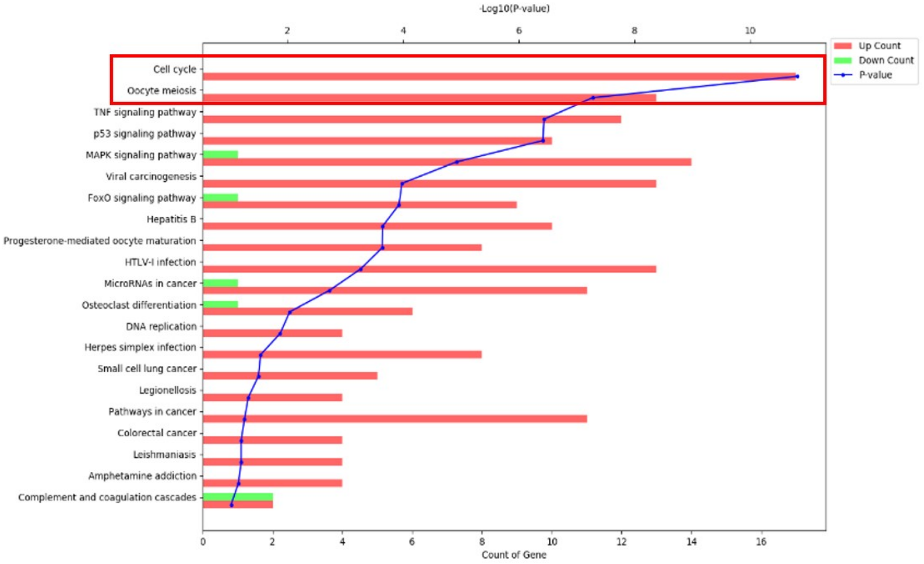

Supplementary Fig. 5. **Gene Ontology (GO) analysis by DAVID database using total 309 differentially expressed genes ('260 upregulated' + '49 downregulated' from ST/ctrl)**. From the top 30 enriched terms of ST versus ctrl are shown in terms of biological process, cellular component, molecular function, and Kyoto Encyclopedia of Genes and Genomes (KEGG). In particular, cell division, an essential biological activity for iPSCs reprogramming, were highlighted by red boxes.

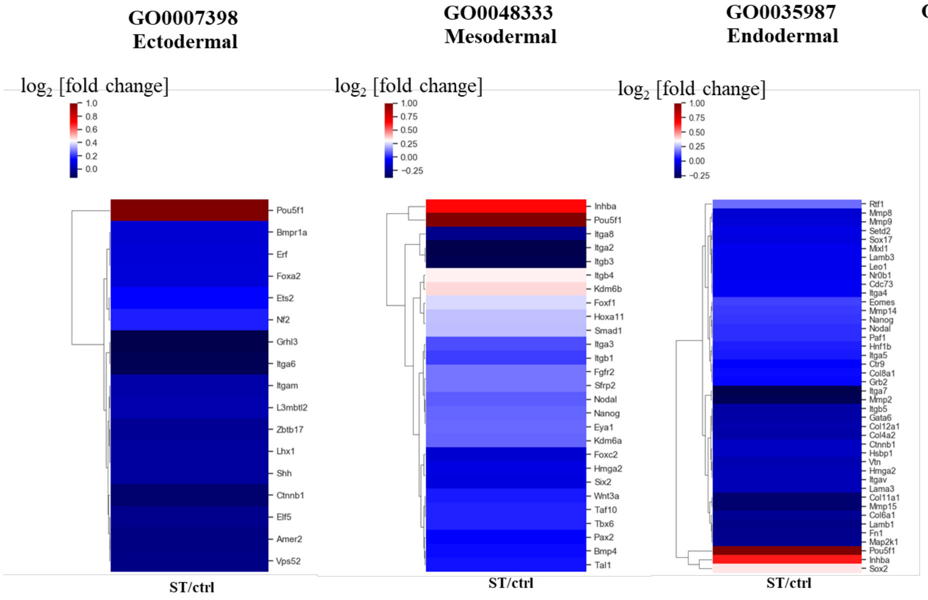

Over 1.5-fold change

Gene symbol	ST/ctrl
Inhba	1.564
Pou5f1	2.183

Supplementary Fig. 6. Gene expression levels related with the differentiation into 3 germ layers at day 2. 3 germ layer related GO (QuickGO, EMBL-EBI) did not reveal significant germ layer specific DEG in ST. They only showed pluripotent markers (Pou5f1 (oct4)) and inhba in ST over 1.5-fold change (same DEG as shown in Fig. 2a).

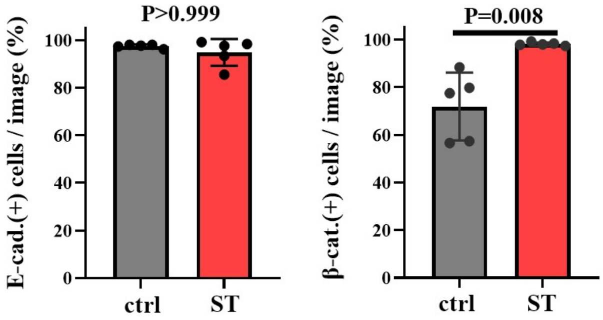

Supplementary Fig. 7. Immunofluorescence-based quantification of the fraction of cells positive for E-cadherin or β-catenin. Non-parametric Mann–Whitney U test. P values are noted in the graphs.

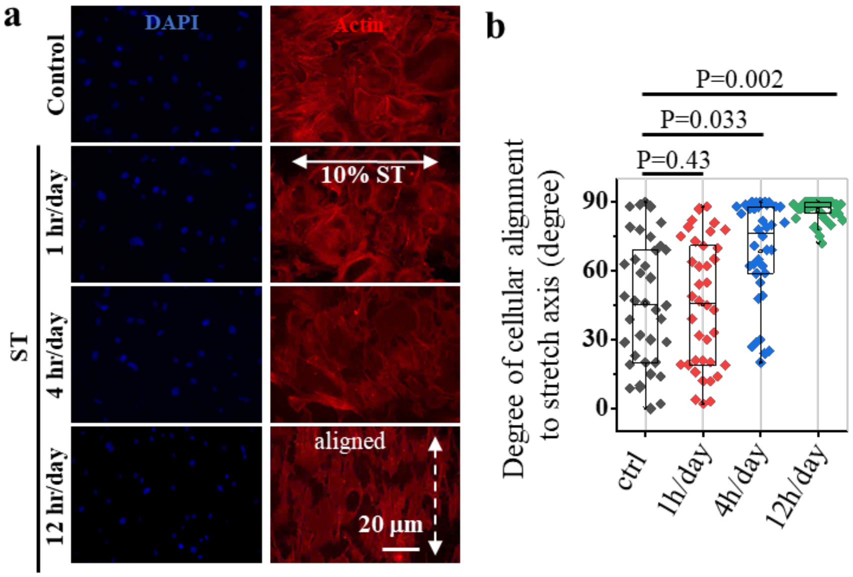

Supplementary Fig. 8. **Degree of cellular alignment, perpendicular to the stretch direction**. (a) Representative cell images and (b) the quantification. Cyclic stretch of a short-period per day (1 h/day) did not induce cellular alignment whereas cyclic stretch of longer periods per day (4 h and 12 h) caused substantial cell alignment (~75° and ~90° on average for 4 h/day and 12 h/day, respectively). The pictures were captured 24 hours post the onset of stretching. Cellular alignment involves the change of F-actin reorganization perpendicular to stretching direction over time. One-way ANOVA and Tukey post hoc test.

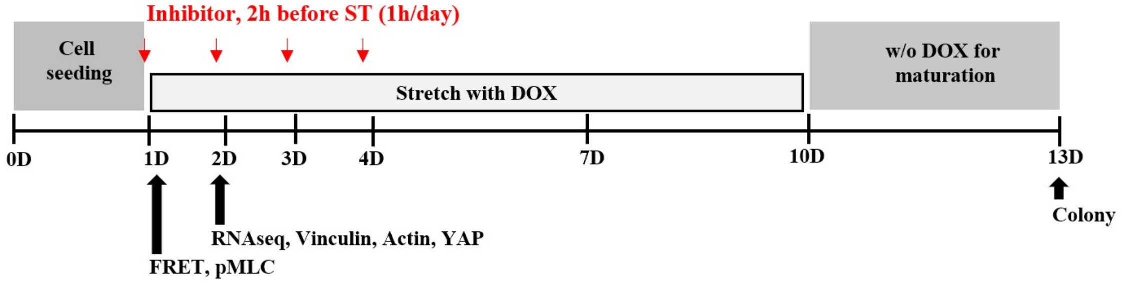

Supplementary Fig. 9. **Experimental scheme, showing the treatment of inhibitors and the analyses of cells**.

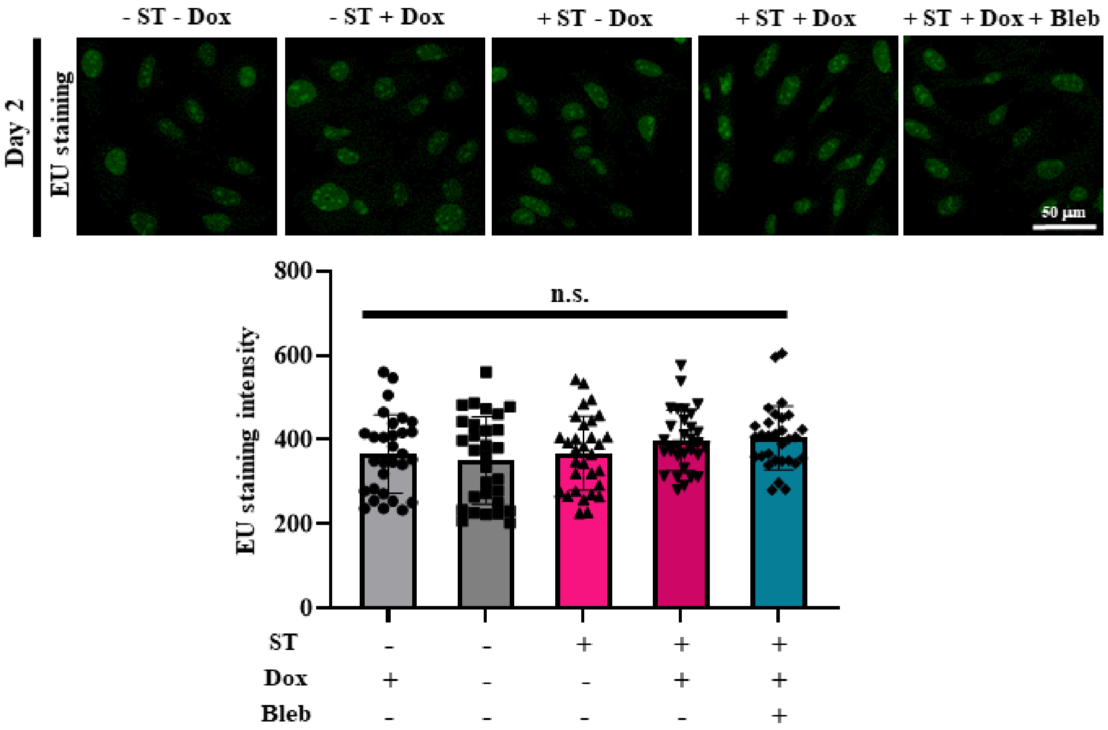

Supplementary Fig. 10. **RNA transcription assay using 5-ethynyl uridine (EU).** Cyclic stretch and inhibitor treatment did not change RNA transcription based on the EU intensity (Bleb is shown representatively). EU was used for fluorescent staining of newly transcribed RNA via metabolic labelling by click chemistry.

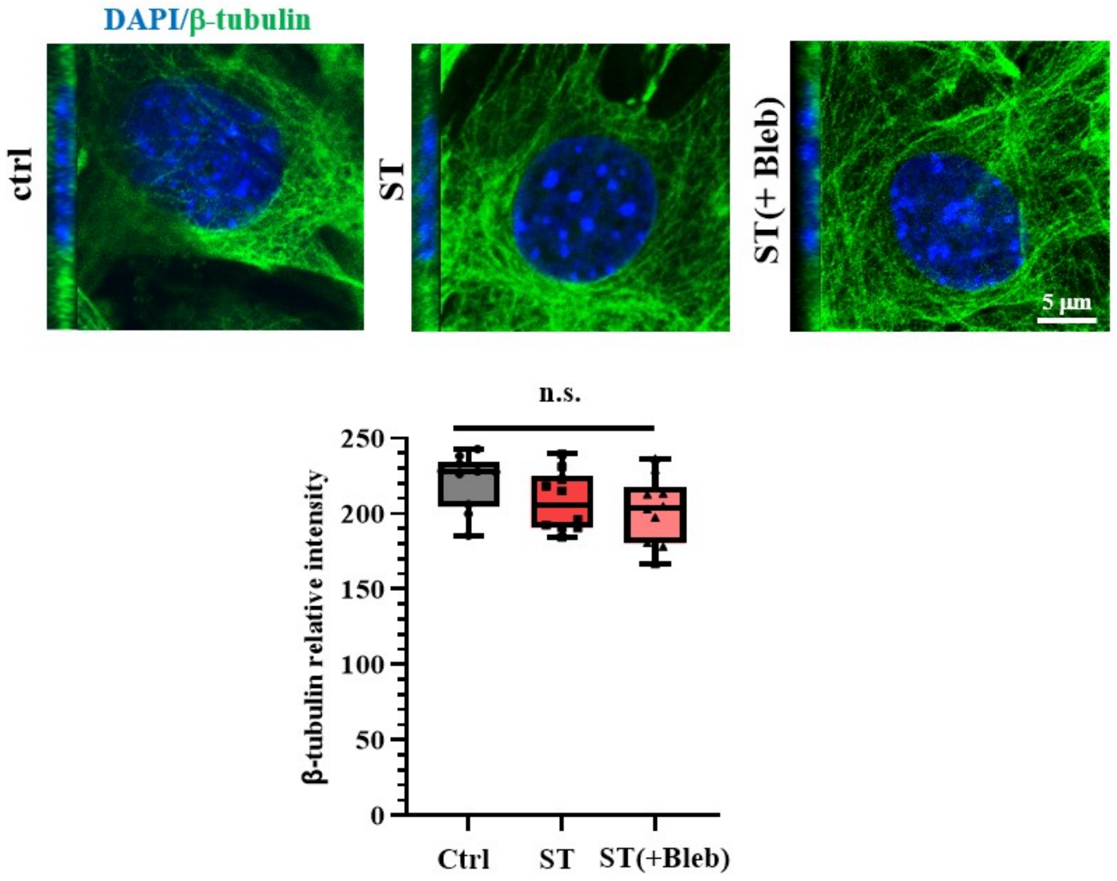

Supplementary Fig. 11. **Expression of β-tubulin.** β-tubulin that comprises microtubules in cytoskeleton was not altered significantly by the cyclic stretch. Non-parametric Kruskal-Wallis test ($P < 0.05$).

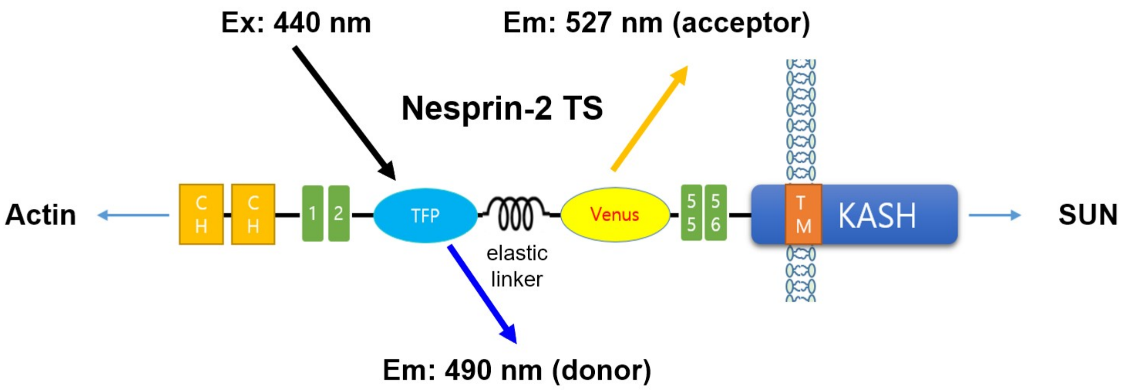

Supplementary Fig. 12. **Schematic of FRET-based nesprin-2 tension sensor (TS).** mTFP (236 amino acids) and Venus (239 amino acids) sequence, separated by an elastic linker of 40 amino acids were inserted between 1-485 N-terminal action binding sequence (including Calponin-homology (CH)1, Calponin-homology (CH)2, Spectrin1, and Spectrin2 domains) and 6525-6874 C-terminal SUN binding sequence (including Spectrin55, Spectrin56, KASH domain) of mouse nesprin-2.

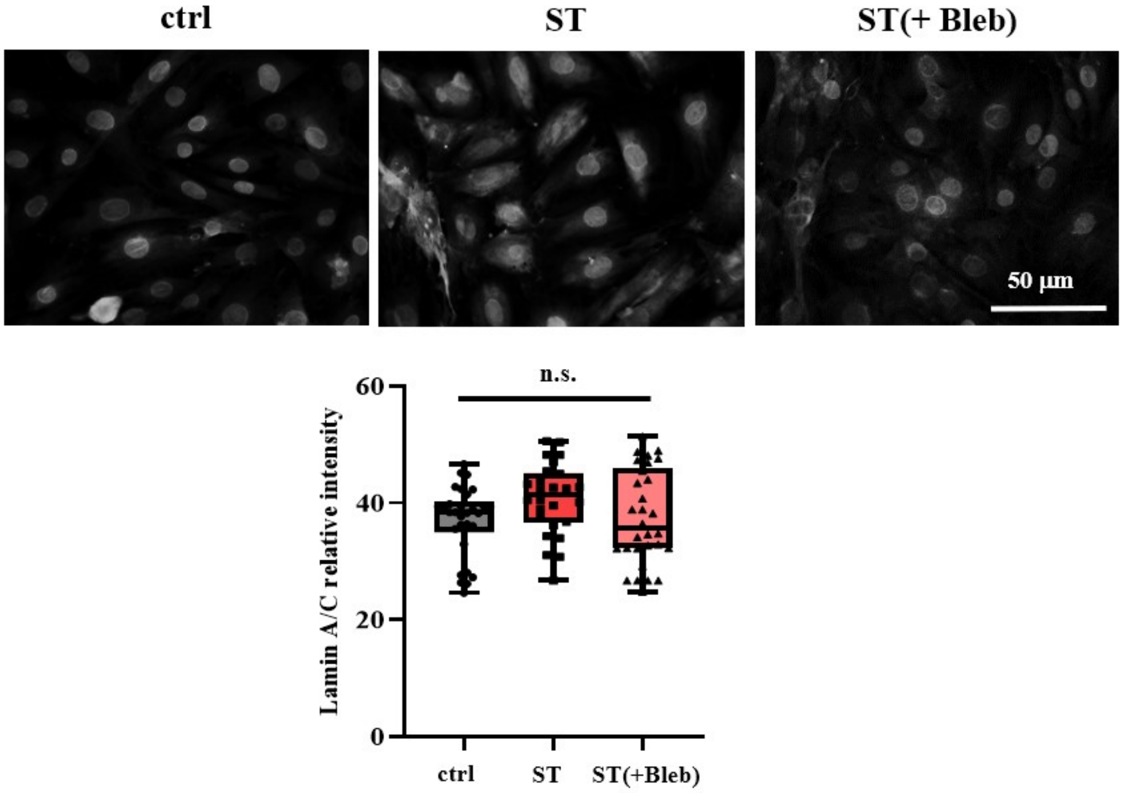

Supplementary Fig. 13. **Expression of Lamin A/C**. Lamin A/C expression in nucleus was not affected significantly by the cyclic stretch. Non-parametric Kruskal-Wallis test ($P < 0.05$).

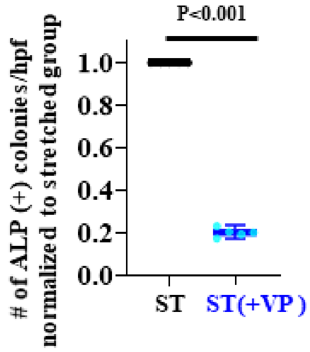

Supplementary Fig. 14. **Effect of YAP inhibition (with VP treatment) on ALP(+) colony formation.** YAP inhibition significantly reduced the level down to ~20% of stretched group (n = 5). Inhibitions of other mechano-machineries are also compared, replotting the data of ST(+VP) after normalization to ST from Fig. 3g.

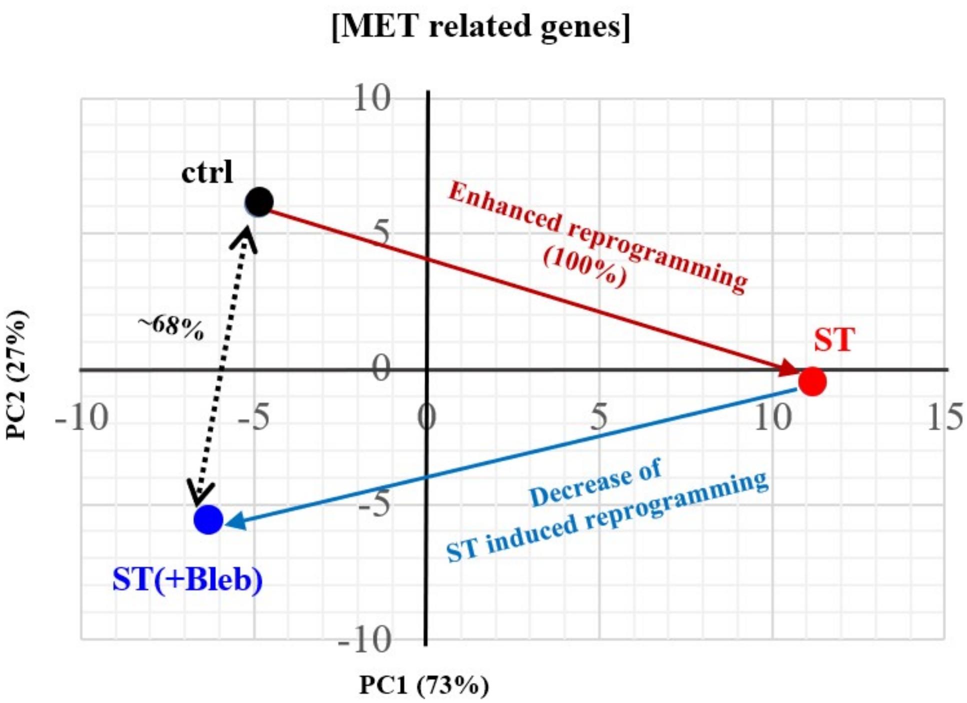

Supplementary Fig. 15. **Principal component analysis (PCA) of the 54 genes that differentially expressed among groups by RNA-sequencing**. 2D-PCA plot, consisting of 73% and 27% for PC1 and PC2 component, respectively, revealed that Bleb treatment group is relatively close to control (w/o stretch) group, indicating a partial recovery (~68%) of transcriptional distance even in the off-target effect of inhibitor.

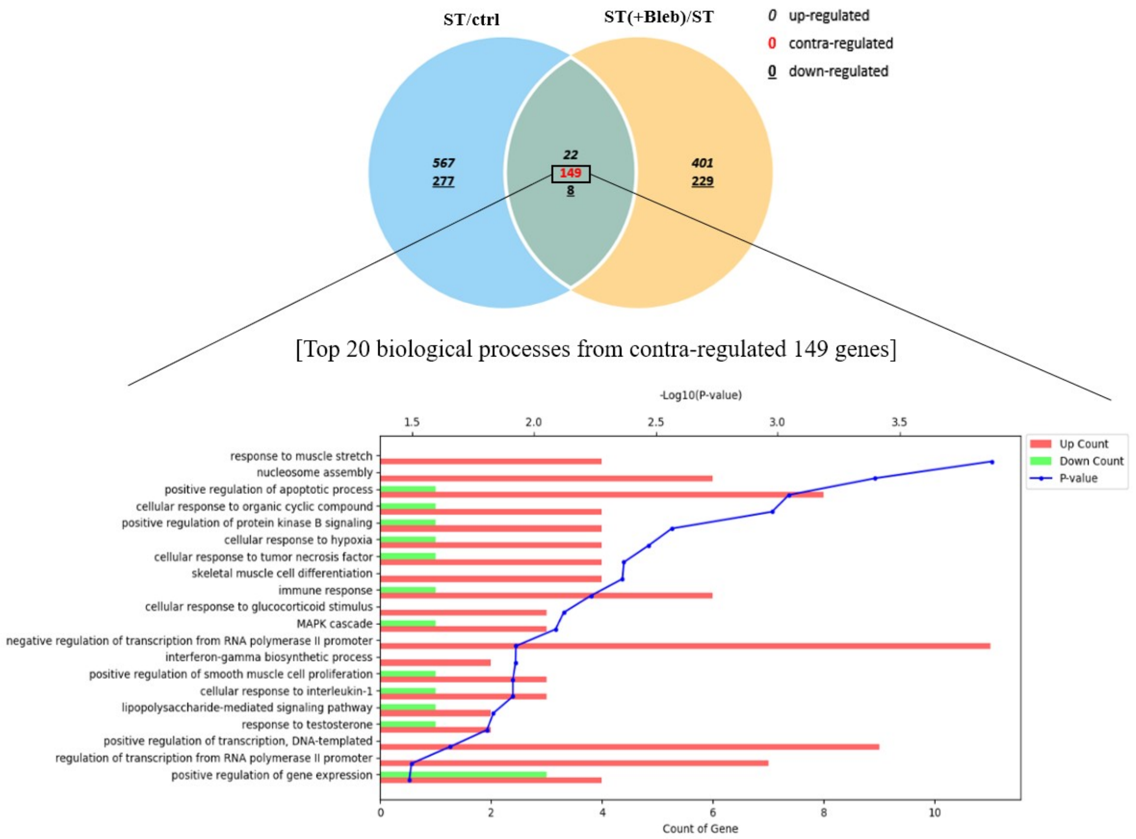

Supplementary Fig. 16. **Venn diagram of differentially expressed genes (DEGs) between ST/ctrl and ST(+Bleb)/ST).** It revealed 22 genes up-regulated, 8 genes down-regulated, and 149 genes contra-regulated, and the top 20 biological processes identified from GO term analysis of the contra-regulated genes.

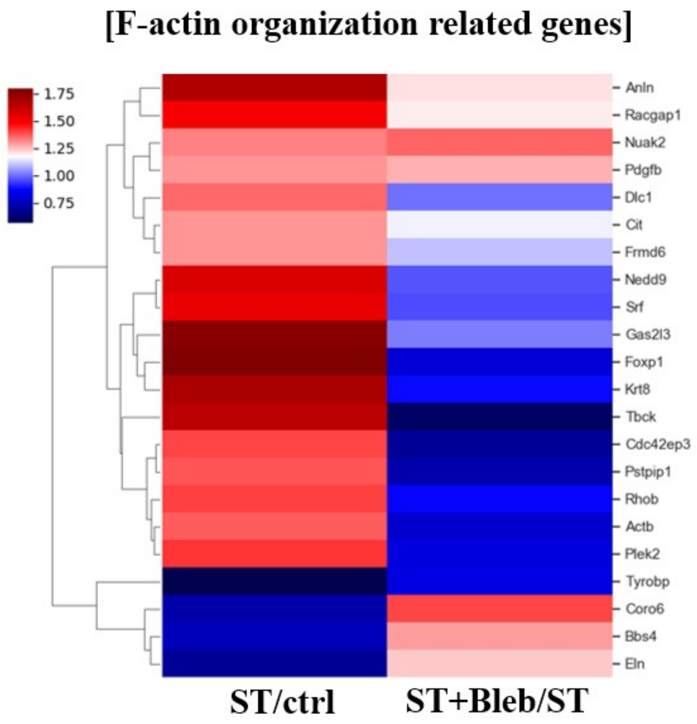

Supplementary Fig. 17. **Transcriptome change (over 1.3-fold) related with F-actin organization.** Widespread up-regulation by the stretch was reversed with blebbistatin treatment.

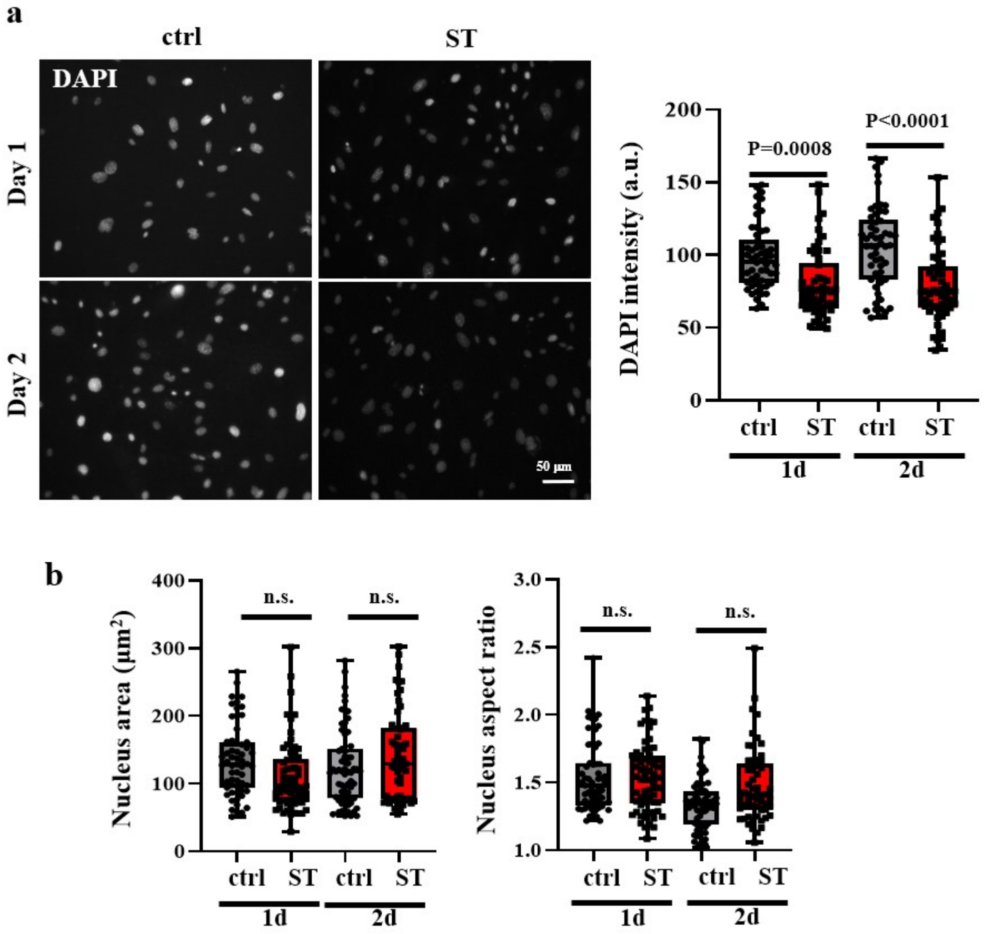

Supplementary Fig. 18. DAPI intensity decreased after cyclic stretch. (**a**) DAPI expression was visualized by fluorescent images at day 1 and 2, and the intensity was quantified to show significant decrease by the stretch (1 h/day). T-test was performed in each day between without and with stretching at a level of given value above (at least 0.05). (**b**) The difference in nucleus area and aspect ratio were not significant (n.s.) by T-test ($P<0.05$).

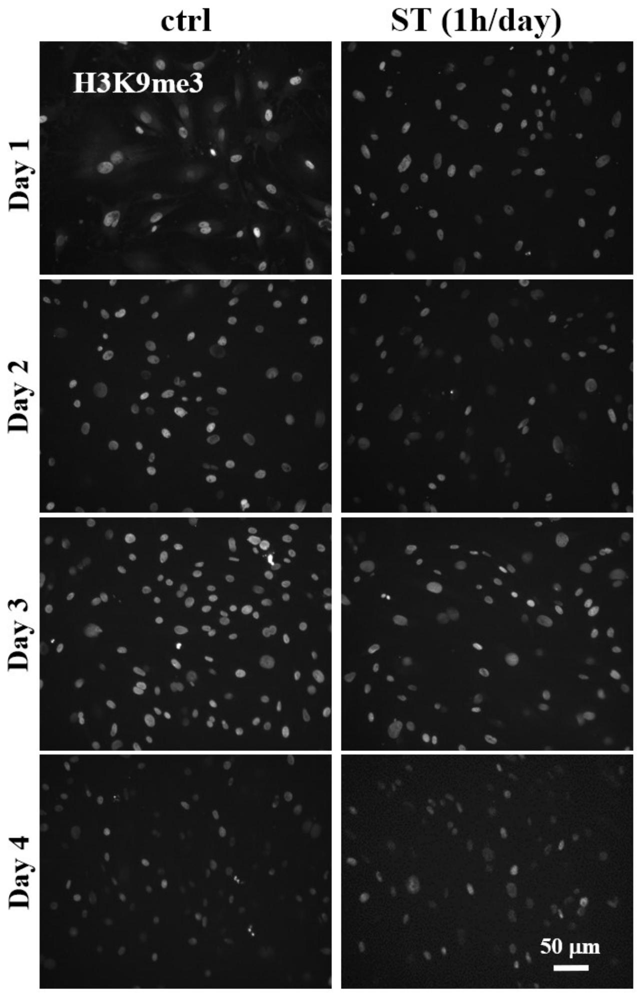

Supplementary Fig. 19. **Representative images of H3K9me3 expression over initial 4 days of reprograming.** H3K9me3 expression visualized by fluorescence images, during cellular reprogramming over the culture period of 4 days. Stretch was for 1 h per day.

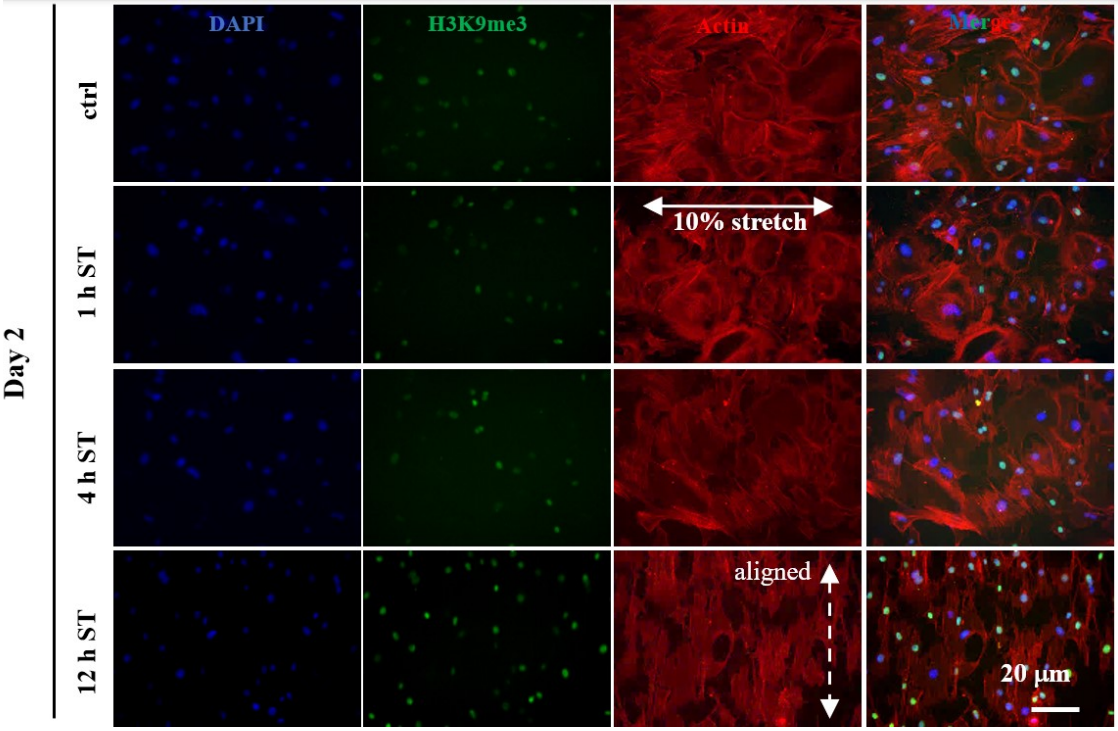

Supplementary Fig. 20. **Effect of stretch duration applied daily (hour per day) on the change in H3K9me3 expression**. DAPI and actin were co-stained. H3K9me3 expression was down-regulated only in 1 h/day (not in extended hours) of stretch, when the cellular alignment was minimally affected. Dapi and actin images were reused from **Supplementary Fig. 8a.**

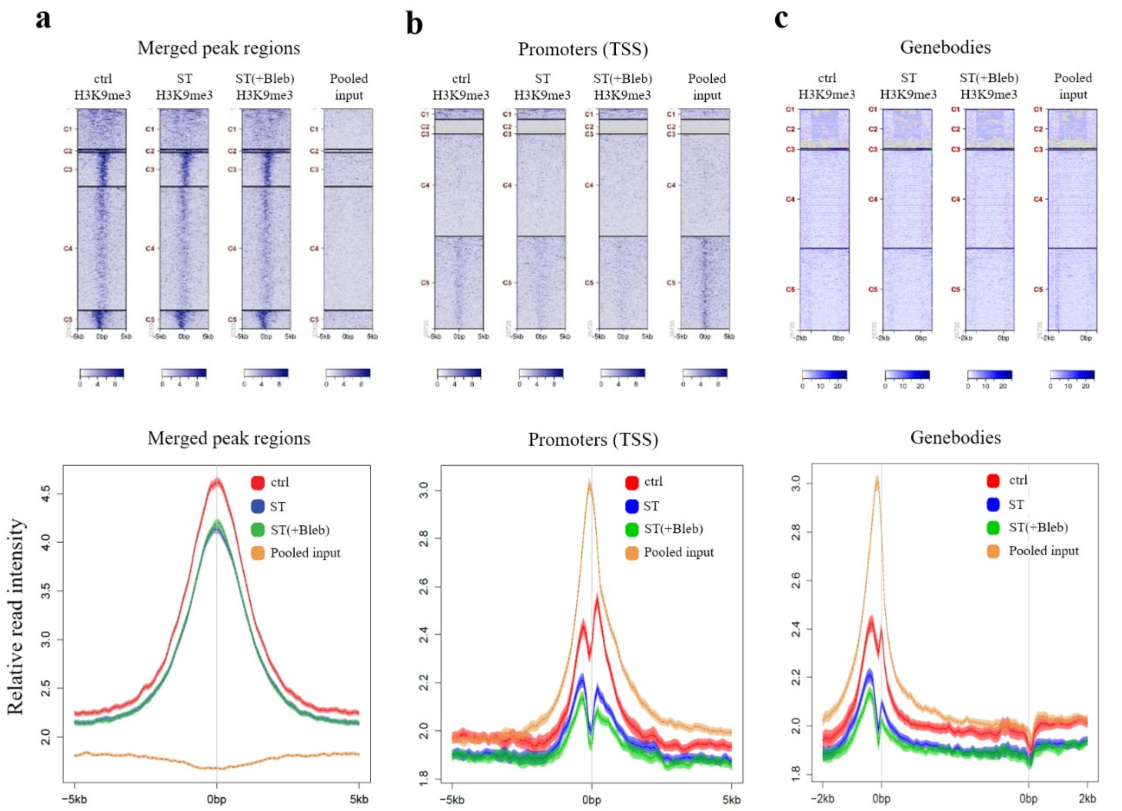

Supplementary Fig. 21. **Occupancy of H3K9me3 in merged peak regions, promoters, and gene bodies by H3K9me3 ChIP-seq analysis**. All three analyzed samples revealed high intensity in (**a**) merged peak regions, (**b**) promoters and (**c**) gene bodies while pooled input, experimental DNA control without H3K9me3-pulled-down, showed no peak in merged regions and high non-specific peaks in promotor and gene body regions unlike H3K9me3-pulled-down groups, confirming successful ChIP-seq analysis using H3K9me3 antibody.

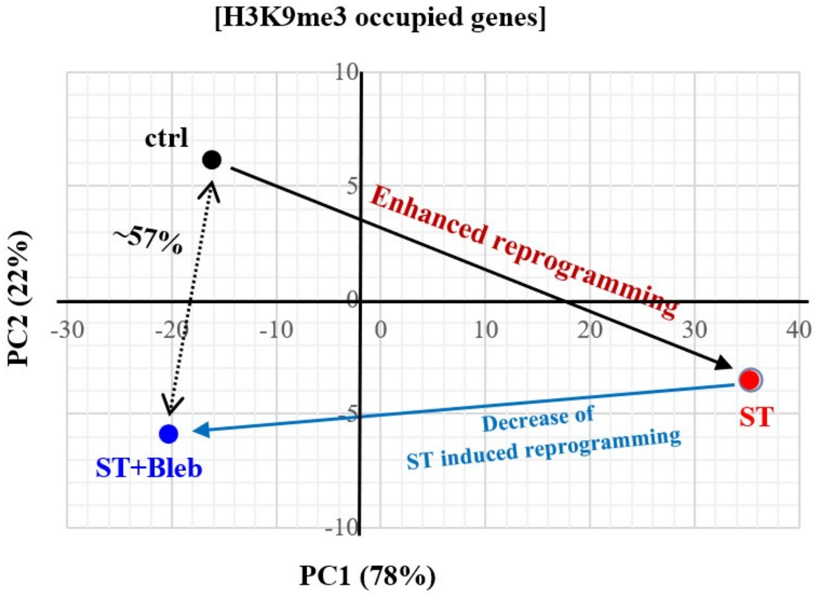

Supplementary Fig. 22. **Principal component analysis (PCA) of the 590 differentially bound regions in H3K9me3 among groups.** 2D-PCA plot, consisting of 78% and 28% for PC1 and PC2 component, respectively, revealed that Bleb treatment group is relatively close to control.

Molecular function

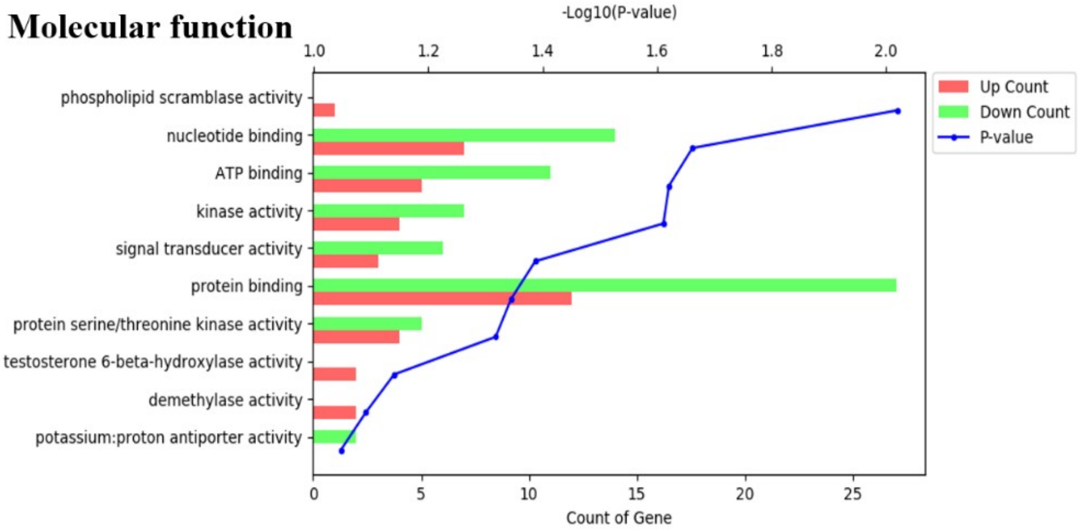

Cell component

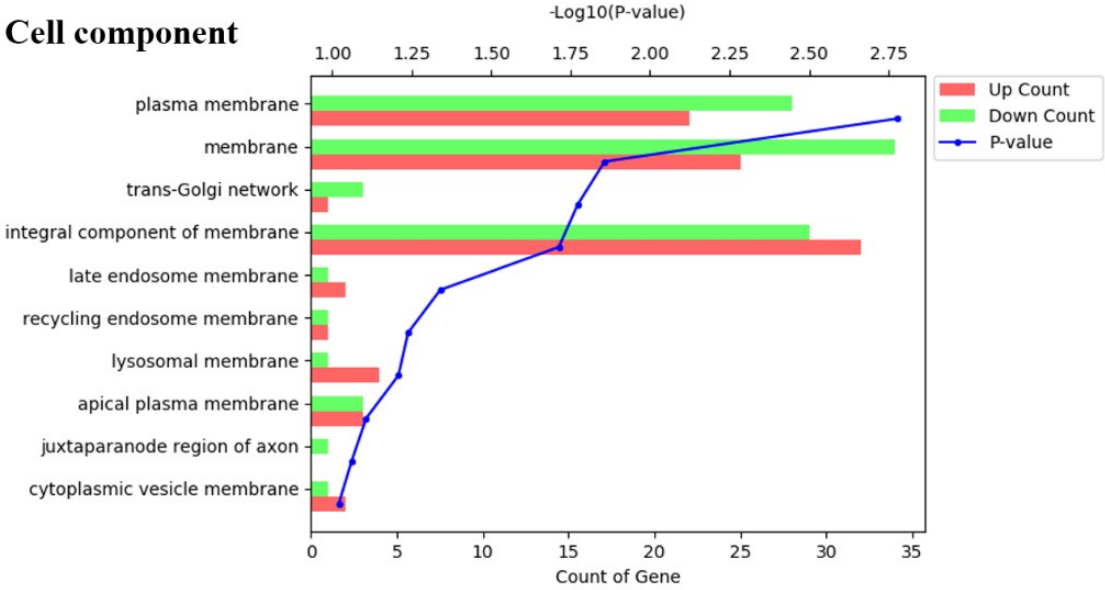

Supplementary Fig. 23. **249 genes from 590 differentially bound regions in H3K9me3 were utilized to perform GO analysis by DAVID**. Top 10 enriched terms (ST/ctrl vs ST+Bleb/ST) related with molecular function and cell component are shown.

a

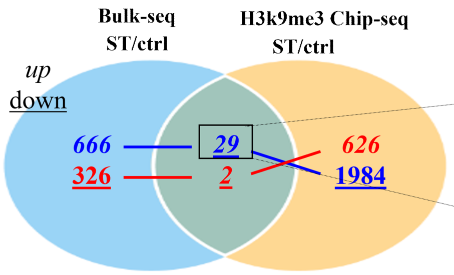

[Bulk-seq up & H3k9me3 Chip-seq down]

Zbtb38 Trim24 Zfhx3 Prss22 Fgf2 Tfeb Wrb Smim3 Zfp36l1 Depdc1b Kifc5b Rasl11a E030011O05Rik Mdm2 Depdc1a Msh2 Mad2l1 Cit Hmgb1 Hnrnpd Anln Acot6 Diap3 Lif Cyp4f39 Mms22l Ankrd1 Igsf3 Bcor

b

<Biological process> * Cell division * Mechanotransduction * Pluripotency

Sublist	Category		Term	RT	Genes	Count	%	P-Value	Benjamini
☐	GOTERM_BP_DIRECT	* (blue)	positive regulation of transcription from RNA polymerase II promoter	RT		7	25.9	2.9E-3	7.1E-1
☐	GOTERM_BP_DIRECT	* (blue)	regulation of cell cycle	RT		4	14.8	5.1E-3	7.1E-1
☐	GOTERM_BP_DIRECT	* (blue)	positive regulation of gene expression	RT		5	18.5	5.4E-3	7.1E-1
☐	GOTERM_BP_DIRECT	* (blue)	cell cycle	RT		5	18.5	6.8E-3	7.1E-1
☐	GOTERM_BP_DIRECT		negative regulation of transcription, DNA-templated	RT		5	18.5	7.8E-3	7.1E-1
☐	GOTERM_BP_DIRECT		lung development	RT		3	11.1	1.1E-2	8.2E-1
☐	GOTERM_BP_DIRECT		regulation of gene expression	RT		4	14.8	1.9E-2	9.5E-1
☐	GOTERM_BP_DIRECT		3'-UTR-mediated mRNA destabilization	RT		2	7.4	2.0E-2	9.5E-1
☐	GOTERM_BP_DIRECT	* (blue)	positive regulation of MAPK cascade	RT		3	11.1	2.3E-2	9.5E-1
☐	GOTERM_BP_DIRECT		cellular response to DNA damage stimulus	RT		4	14.8	2.4E-2	9.5E-1
☐	GOTERM_BP_DIRECT		positive regulation of vascular endothelial cell proliferation	RT		2	7.4	2.5E-2	9.5E-1
☐	GOTERM_BP_DIRECT		negative regulation of blood vessel endothelial cell migration	RT		2	7.4	2.6E-2	9.5E-1
☐	GOTERM_BP_DIRECT	* (red)	protein localization to chromatin	RT		2	7.4	2.8E-2	9.5E-1
☐	GOTERM_BP_DIRECT		positive regulation of sprouting angiogenesis	RT		2	7.4	2.9E-2	9.5E-1
☐	GOTERM_BP_DIRECT		negative regulation of transcription from RNA polymerase II promoter	RT		5	18.5	3.2E-2	9.6E-1
☐	GOTERM_BP_DIRECT		regulation of mRNA stability	RT		2	7.4	4.0E-2	1.0E0
☐	GOTERM_BP_DIRECT	* (blue)	endothelial cell proliferation	RT		2	7.4	4.1E-2	1.0E0
☐	GOTERM_BP_DIRECT	* (blue)	mitotic sister chromatid segregation	RT		2	7.4	4.3E-2	1.0E0
☐	GOTERM_BP_DIRECT	* (blue)	positive regulation of mitotic cell cycle	RT		2	7.4	4.5E-2	1.0E0
☐	GOTERM_BP_DIRECT		positive regulation of blood vessel endothelial cell migration	RT		2	7.4	5.0E-2	1.0E0
☐	GOTERM_BP_DIRECT		heart development	RT		3	11.1	5.1E-2	1.0E0
☐	GOTERM_BP_DIRECT		positive regulation of vascular smooth muscle cell proliferation	RT		2	7.4	5.1E-2	1.0E0
☐	GOTERM_BP_DIRECT	* (blue)	positive regulation of transcription, DNA-templated	RT		4	14.8	5.5E-2	1.0E0
☐	GOTERM_BP_DIRECT		blood vessel remodeling	RT		2	7.4	5.9E-2	1.0E0
☐	GOTERM_BP_DIRECT	* (blue)	cell proliferation	RT		3	11.1	6.5E-2	1.0E0
☐	GOTERM_BP_DIRECT	* (blue)	ERK1 and ERK2 cascade	RT		2	7.4	6.7E-2	1.0E0
☐	GOTERM_BP_DIRECT	* (red)	chromatin organization	RT		3	11.1	7.0E-2	1.0E0
☐	GOTERM_BP_DIRECT	* (blue)	mitotic cytokinesis	RT		2	7.4	7.1E-2	1.0E0
☐	GOTERM_BP_DIRECT		protein kinase B signaling	RT		2	7.4	7.8E-2	1.0E0
☐	GOTERM_BP_DIRECT		DNA repair	RT		3	11.1	7.8E-2	1.0E0
☐	GOTERM_BP_DIRECT	* (green)	stem cell differentiation	RT		2	7.4	7.9E-2	1.0E0
☐	GOTERM_BP_DIRECT		negative regulation of gene expression	RT		3	11.1	7.9E-2	1.0E0
☐	GOTERM_BP_DIRECT	* (blue)	cell division	RT		3	11.1	8.0E-2	1.0E0
☐	GOTERM_BP_DIRECT		cellular response to transforming growth factor beta stimulus	RT		2	7.4	9.0E-2	1.0E0
☐	GOTERM_BP_DIRECT		negative regulation of cell proliferation	RT		3	11.1	9.3E-2	1.0E0

<Pathway>

Sublist	Category		Term	RT	Genes	Count	%	P-Value	Benjamini
☐	KEGG_PATHWAY	* (green)	Signaling pathways regulating pluripotency of stem cells	RT		3	11.1	7.7E-3	3.8E-1
☐	KEGG_PATHWAY		Melanoma	RT		2	7.4	6.8E-2	1.0E0
☐	KEGG_PATHWAY		Platinum drug resistance	RT		2	7.4	7.6E-2	1.0E0
☐	KEGG_PATHWAY		Pathways in cancer	RT		3	11.1	9.5E-2	1.0E0

Sublist	Category		Term	RT	Genes	Count	%	P-Value	Benjamini
☐	WIKIPATHWAYS	* (green)	Mechanisms associated with pluripotency	RT		4	14.8	1.6E-2	2.5E-1
☐	WIKIPATHWAYS	* (green)	ESC pluripotency pathways	RT		3	11.1	2.1E-2	2.5E-1

Sublist	Category		Term	RT	Genes	Count	%	P-Value	Benjamini
☐	REACTOME_PATHWAY	* (red)	RHOB GTPase cycle	RT		3	11.1	4.4E-3	4.5E-1
☐	REACTOME_PATHWAY	* (red)	RHOC GTPase cycle	RT		3	11.1	4.4E-3	4.5E-1
☐	REACTOME_PATHWAY	* (red)	RHOA GTPase cycle	RT		3	11.1	1.7E-2	1.0E0
☐	REACTOME_PATHWAY		Signaling by Rho GTPases	RT		4	14.8	5.1E-2	1.0E0
☐	REACTOME_PATHWAY		Signaling by Rho GTPases, Miro GTPases and RHOBTB3	RT		4	14.8	5.5E-2	1.0E0

Supplementary Fig. 24. Combined analysis of genes upregulated in RNA-seq with reduced occupancy of H3K9me3 in ChIP-seq. (a) 29 genes upregulated in the RNA-seq with reduced occupancy of H3K9me3 (Venn diagram, black box), as analyzed by DAVID, which include **(b)** cell proliferation-, mechanotransduction-, and pluripotency-related GO terms in biological process and pathway.

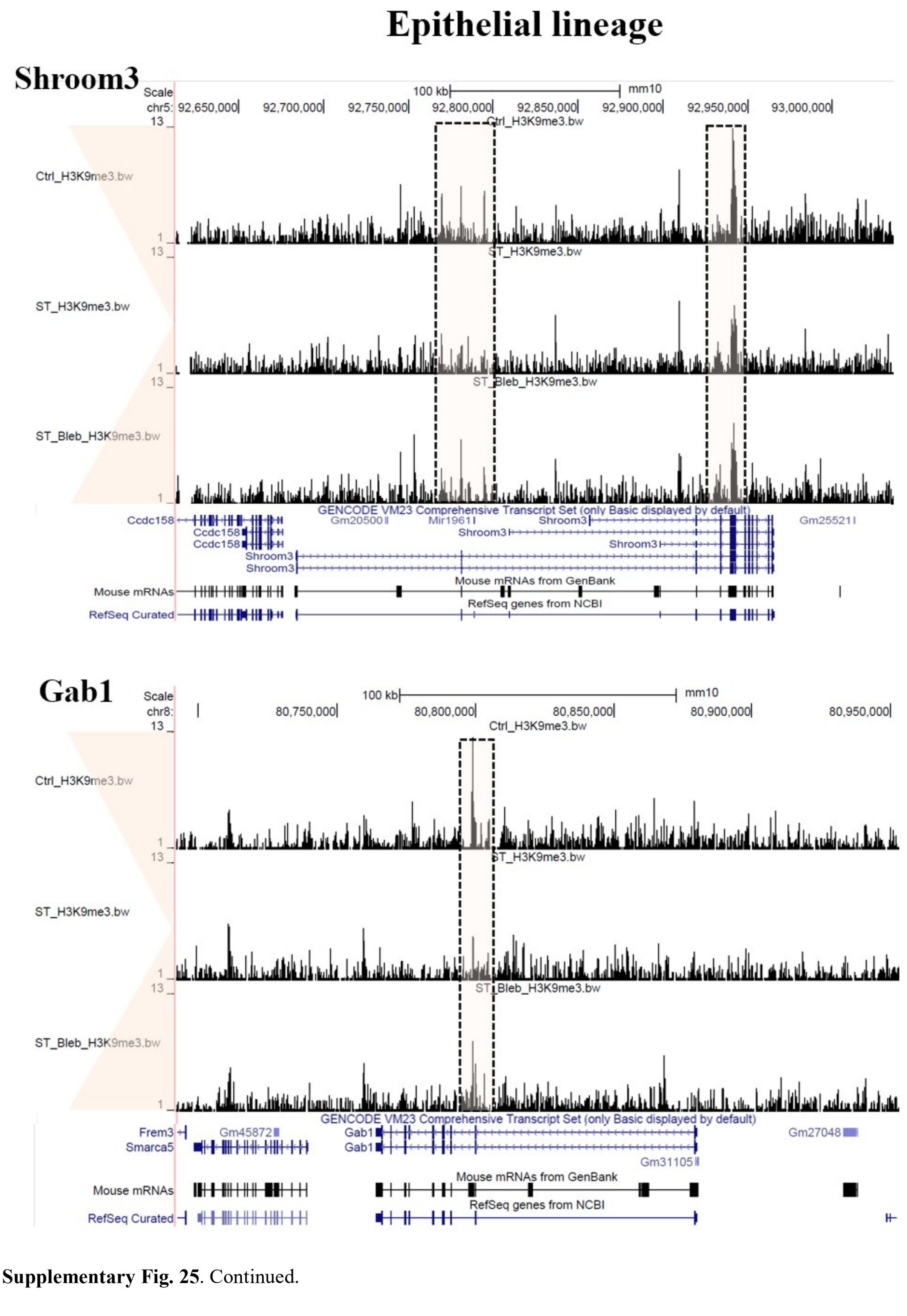

Supplementary Fig. 25. Continued.

Euchromatin

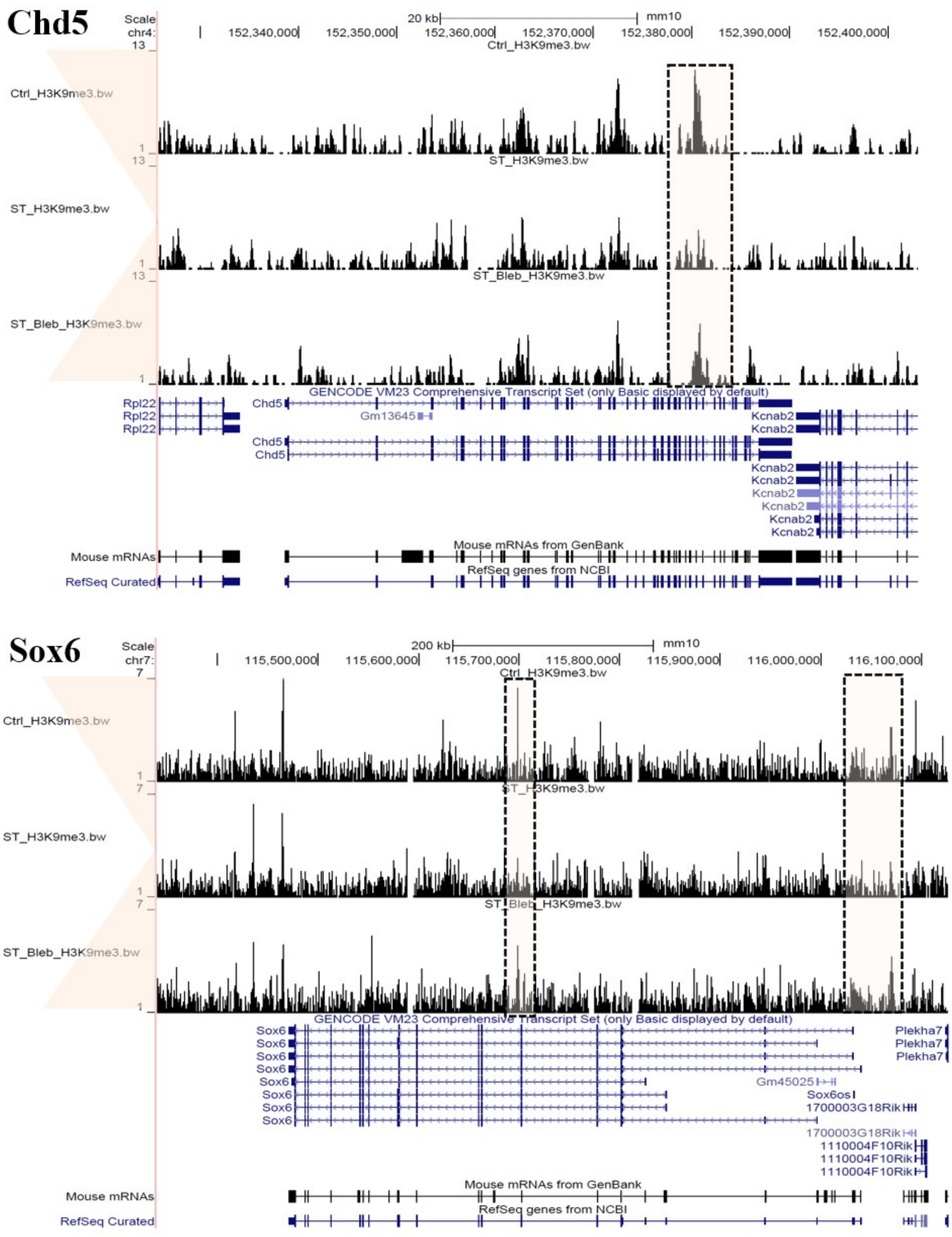

Supplementary Fig. 25. Continued.

Heterochromatin

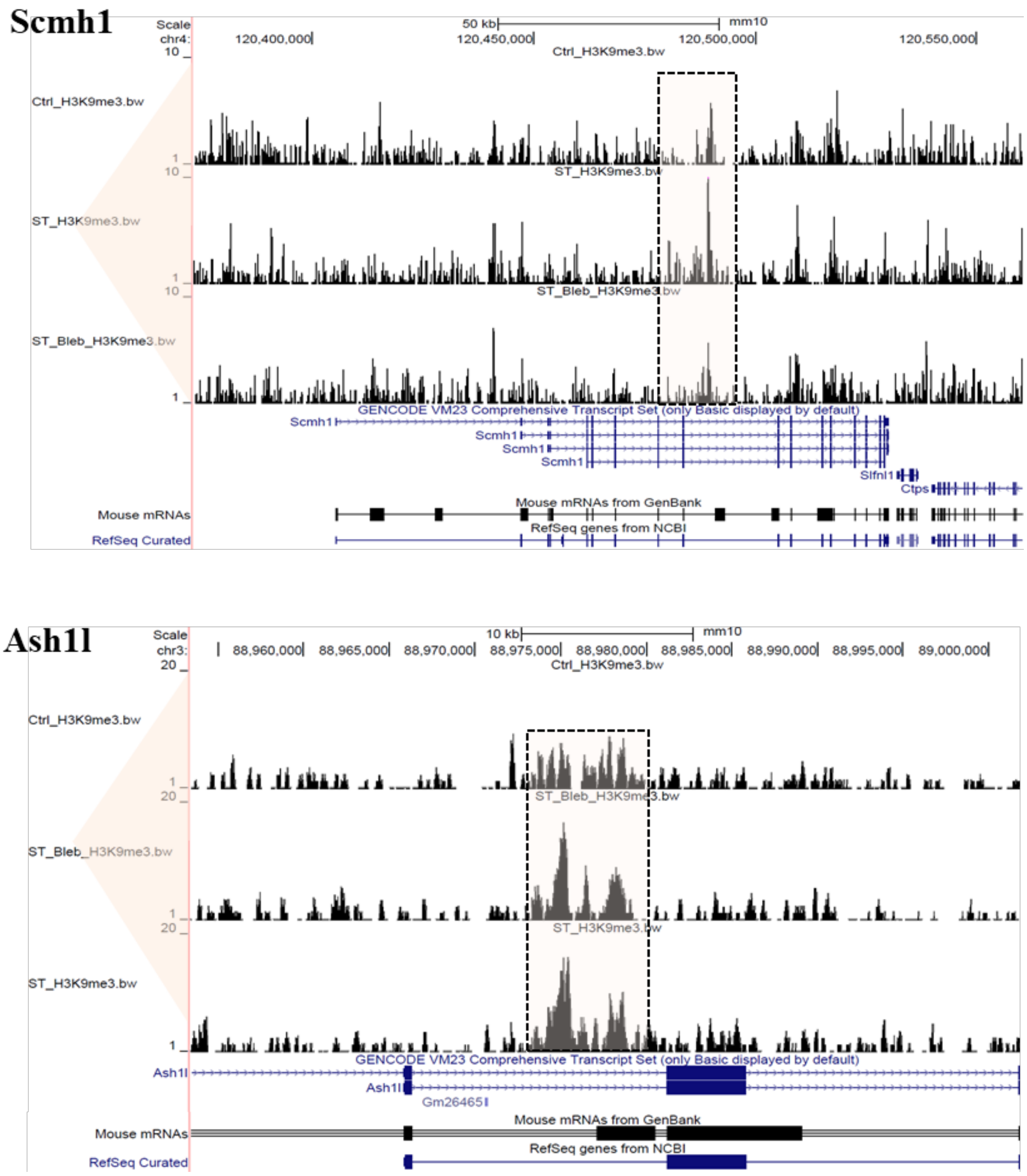

Supplementary Fig. 25. Continued.

Mechanotransduction

Ipo11

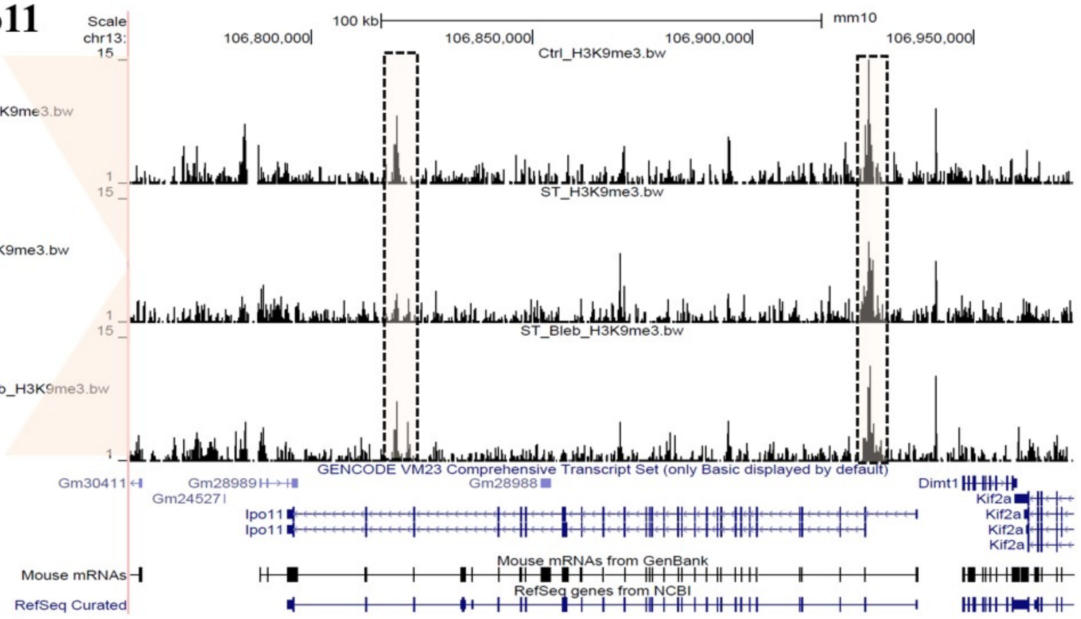

Rapgef4

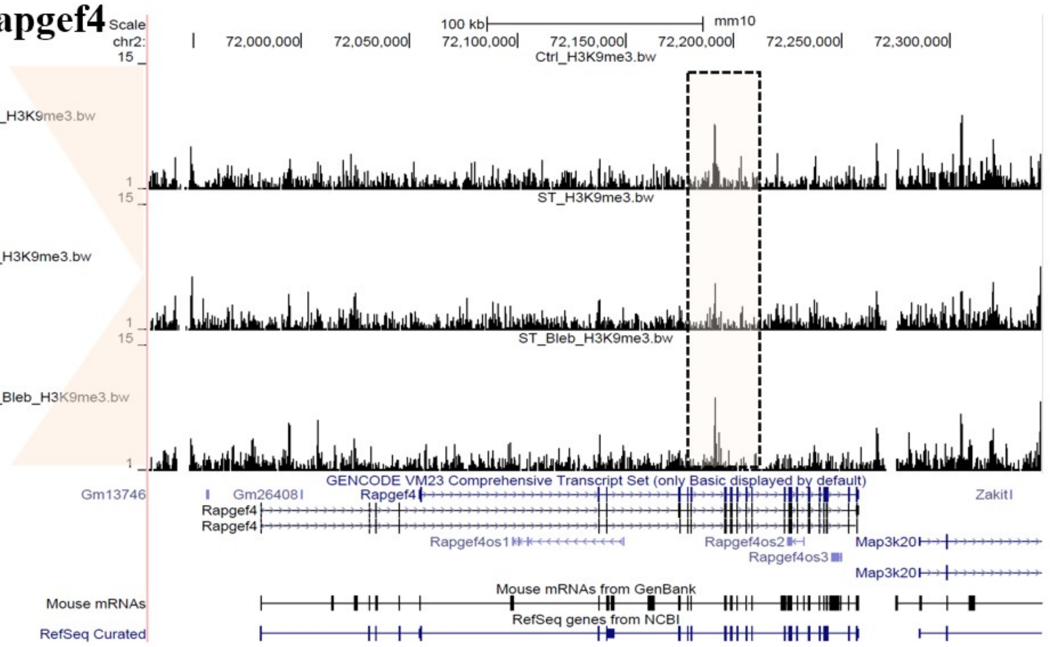

Supplementary Fig. 25. **Representative ChIP-seq reads and peaks via UCSC genome browser.** Stretch-induced decrease in H3K9me3 occupancy was observed (vs. control) at promoters or bodies of genes associated with epithelial lineage (Shroom3 and Gab1), euchromatin (Chd5 and Sox6), heterochromatin (Scmh1 and Ash1l) and mechanotransduction (Ipo11 and Rapgef4), which is partially retrieved by blebbistatin treatment. Reference sequence was shown below in each panel. Peak regions with distinct intensity among groups (fold change >1.5) of four categories from auto peak-calling-algorithm (SICER 1.1, genome mm10) were representatively shown on UCSC genome browser and highlighted as black dot lines. Peak tendency from detected regions among groups in each genome was depicted in y-axis.

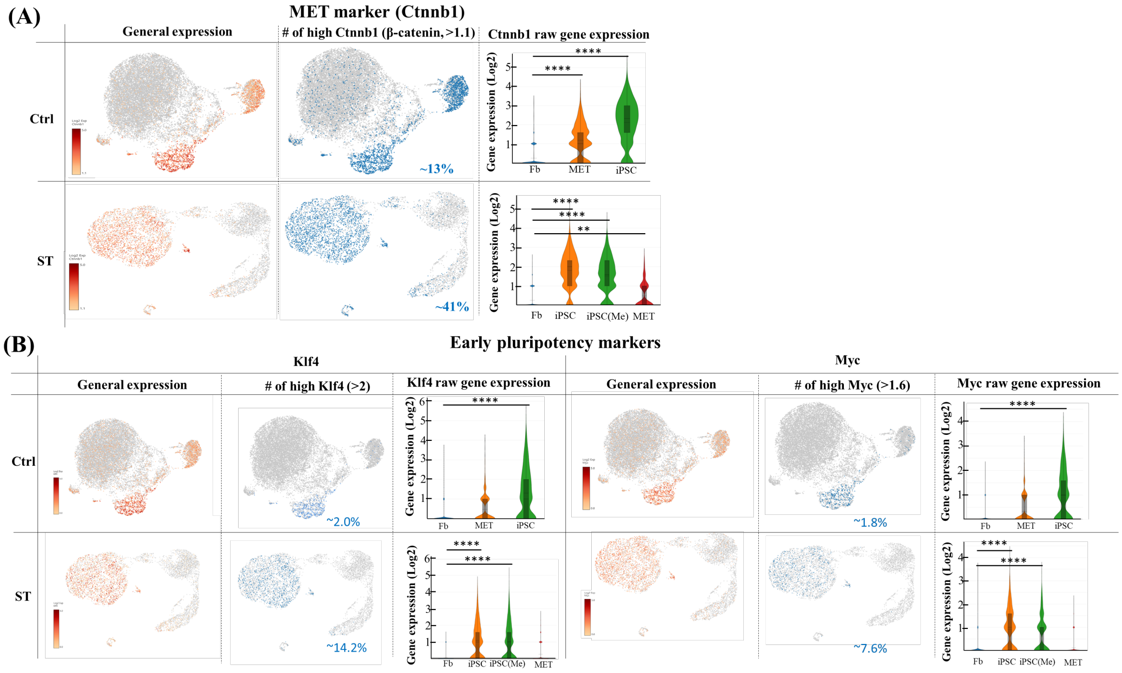

Supplementary Fig. 26. Single cell gene expression of MET marker (Ctnnb1, a) and early pluripotency markers (Klf4 and Myc, b).

Top 50 upregulated genes in iPSC cluster (vs. other clusters)

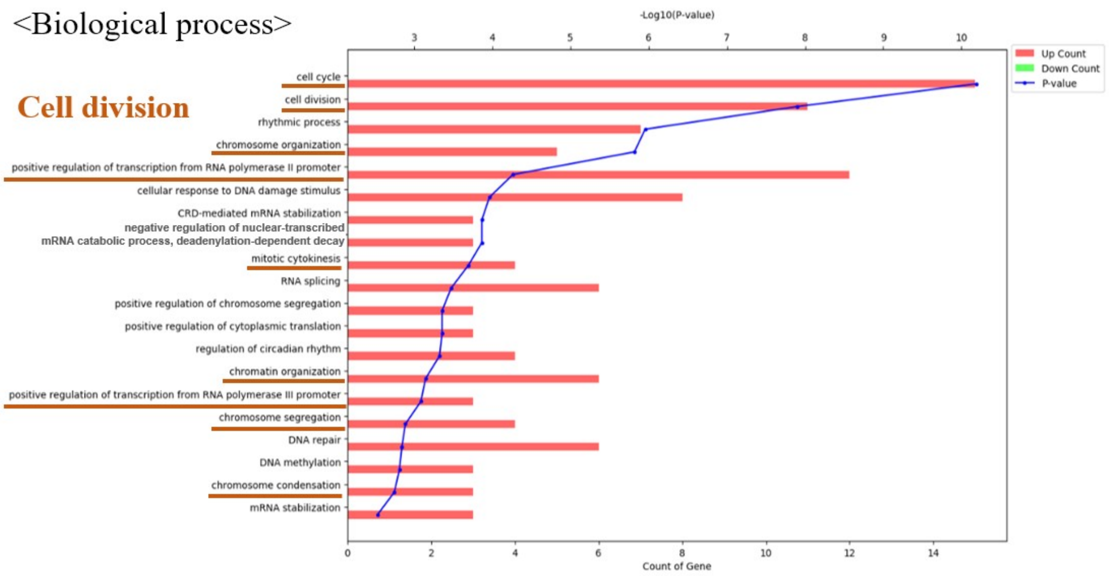

Supplementary Fig. 27. GO analysis of single cell sequencing in ST group. GO analysis by DAVID (biological process) using highly upregulated top 50 genes in iPSC cluster (vs. other clusters) features biological processes related with cell division.

Top 50 upregulated genes in iPSC(ME) cluster (vs. other clusters)

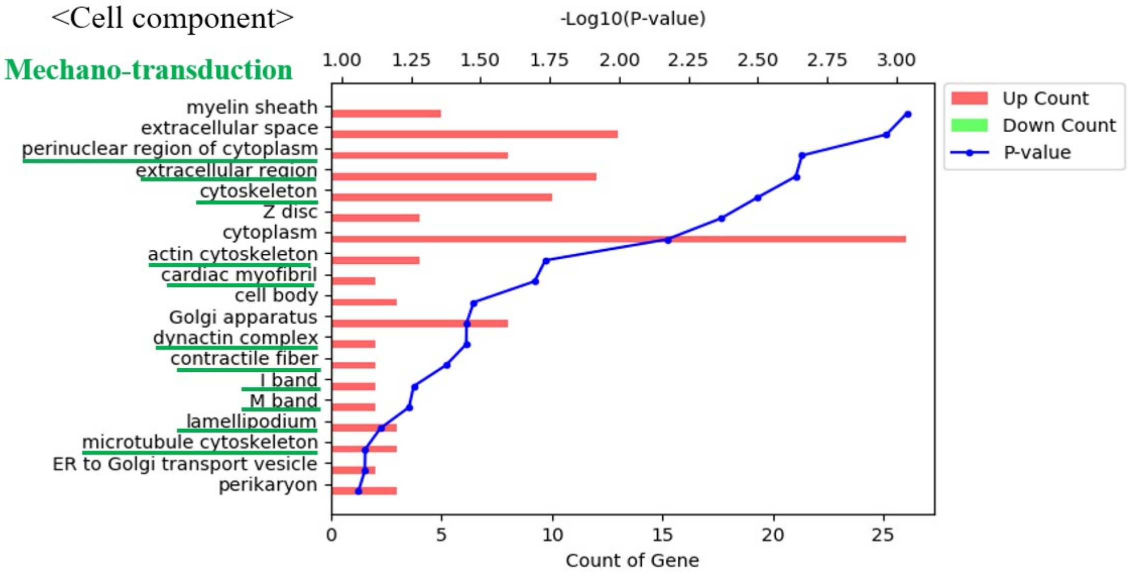

Supplementary Fig. 28. GO analysis of single cell sequencing in ST group. GO analysis by DAVID (cell component) using highly upregulated top 50 genes in iPSC(Me) cluster (vs. other clusters) features those related with mechanotransduction.

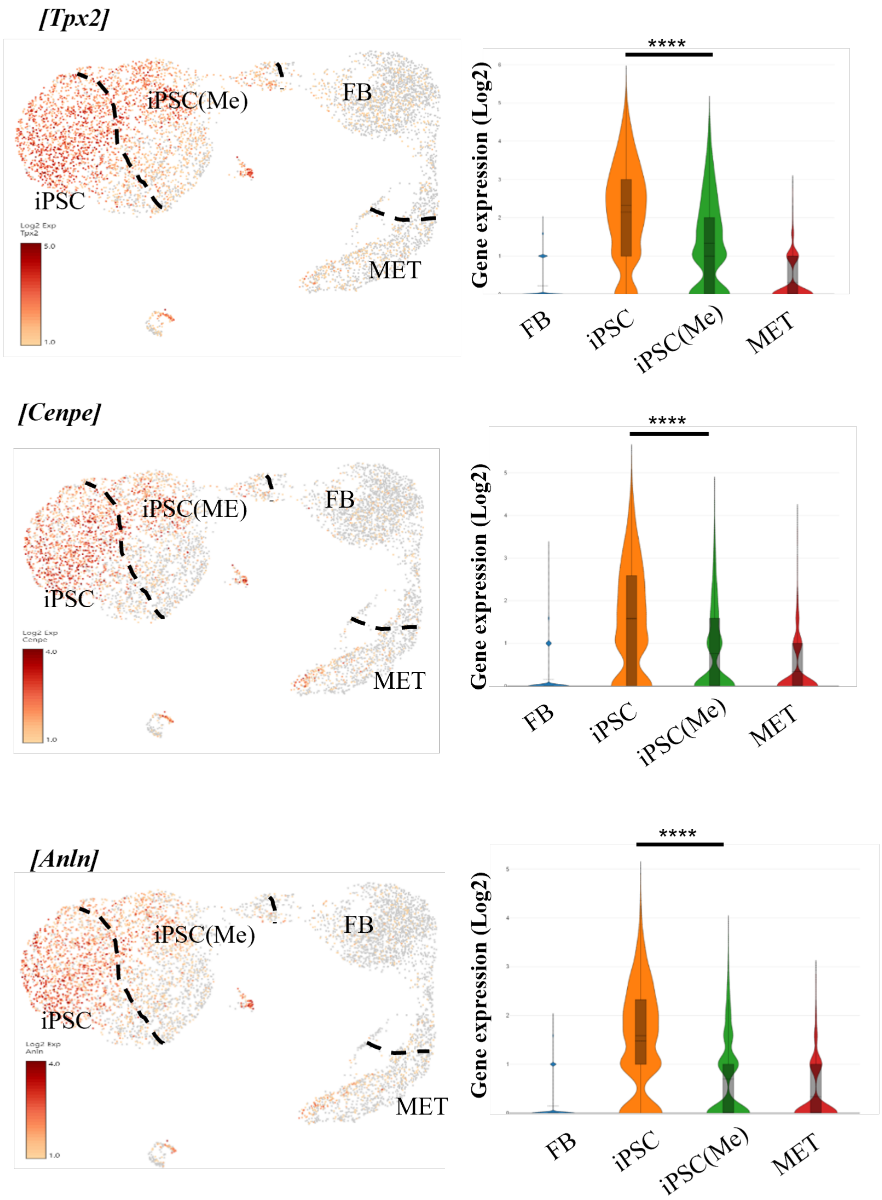

Supplementary Fig. 29. Single cell expression of some representative genes related with cell division in ST group. Tpx2 (one of the many spindle assembly factors), Cenpe (centrosome-associated protein E), and Anln (key in cytoskeletal dynamics during cytokinesis), shows upregulation in iPSC cluster relative to iPSC(Me) cluster.

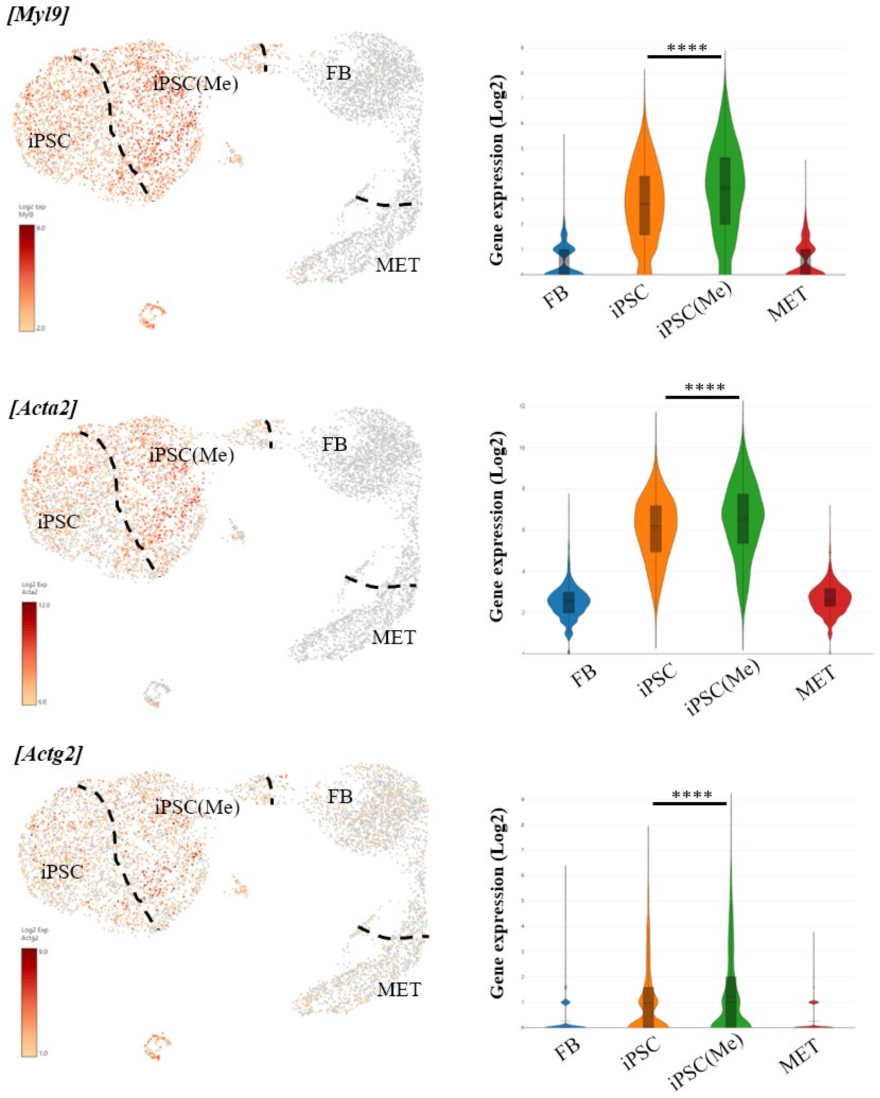

Supplementary Fig. 30. Single cell expression of some representative genes related with mechanotransduction in ST group. Myl9 (myosin light chain protein regulating actomyosin contractility), Acta2 and Actg2 (key actin proteins involved in cellular contractility), shows upregulation in iPSC(Me) cluster relative to iPSC cluster.

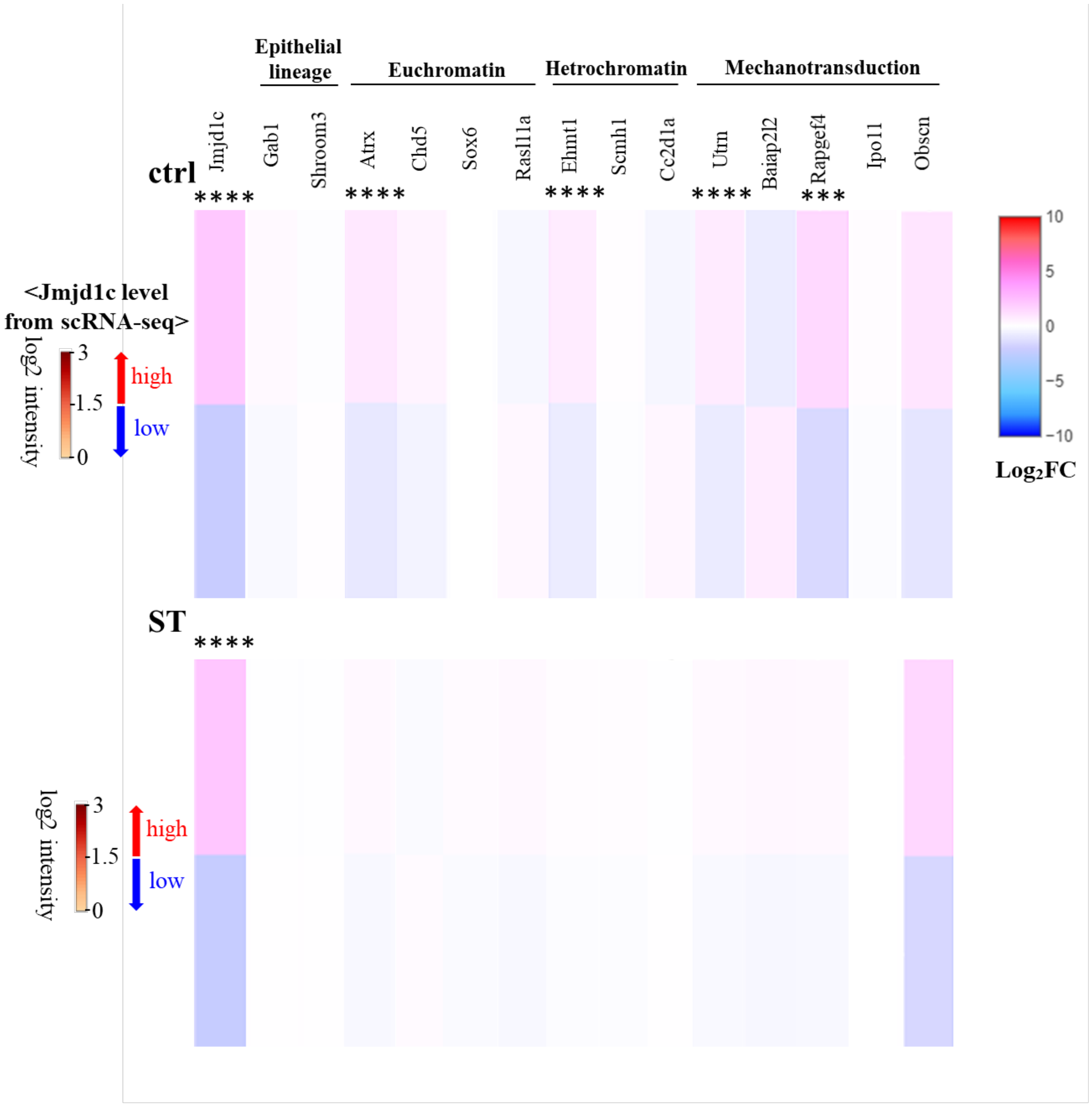

Supplementary Fig. 31. Analyses of Jmjd1c transcription level dependent expression of the genes differentiently occupied by H3K9me3 (in Fig. 5d) by single cell RNA-seq. The cells were divided into high and low expression groups based on a value of 1.5 (using Log2 intensity, refer to Fig. 6c). It revealed Atrx (an euchromatin marker), Utrn and Rapgef4 (mechanotransduction markers) were positively regulated by Jmjd1c (H3K9 demethylase) in ctrl while only slight regulation without significant difference was detected in ST. This lack of significant regulation may be attributed to the higher cell proliferation in ST and consequent fast turn over between gene expression and H3K9me3 occupancy change, partially supporting the role of Jmjd1c in altering genes occupied by H3K9me3. P-value are adjusted using the Benjamini-Hochberg correction for multiple tests between high and low clusters in ctrl and ST respectively (Loupe Browser 6.3.0, ****: $P<0.001$, ***: $P<0.01$).

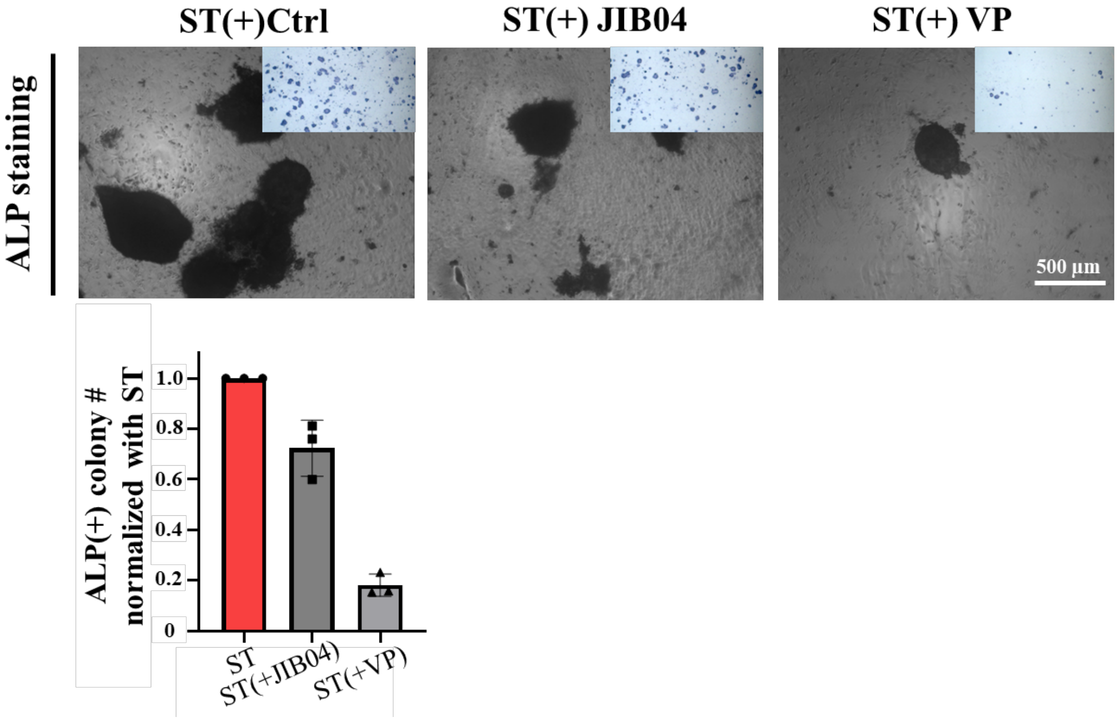

Supplementary Fig. 32 The number of ALP(+) colonies stimulated by a cyclic stretch was diminished by epigenetic or mechanotransductive inhibitors. JIB04 (Jmjd1c inhibitor) and VP (Yap inhibitor) was individually treated during reprogramming process. ALP stained images were shown with its quantification. Inhibitory effects of VP (~80% reduction) is more prominent than JIB04 (20~40% reduction). Data was shown after normalization for each stretching group independently.

a

Bulk RNA-seq

[from histone methyltransferase]

Gene symbol	ST/ctrl
Chtop	1.021
Dot1l	1.828
Ehmt1	0.979
Ezh1	0.957
Ezh2	1.333
Nsd1	1.023
Prmt2	0.776
Prmt6	1.042
Set	1.056
Setbp1	1.207
Setd1b	1.739
Setd4	0.807
Setd5	1.141
Setmar	0.904
Suv39h1	1.176
Suv420h1	1.131

Color scale: -3, -1, -0.33

b

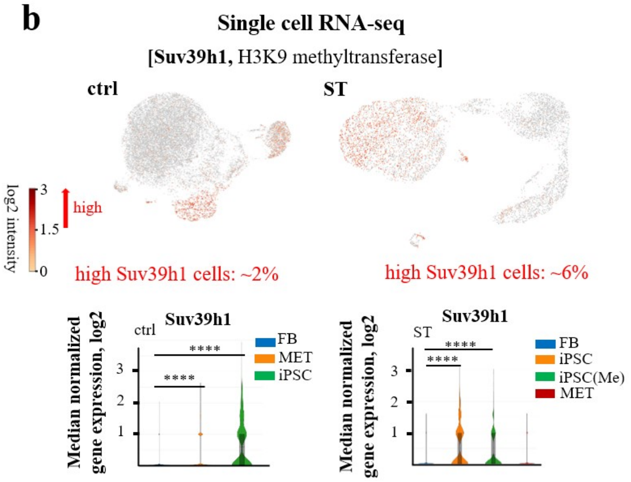

Supplementary Fig. 33. Bulk and single cell RNA-seq analyses of histone methyltransferases, especially Suv39h1. (a) Bulk RNA-seq reveals only a few DEGs in histone methyltransferase. The change in expression of Suv39h1, particularly considered as an important methyltransferase responsible for H3K9me3 and the formation of heterochromatin in iPSCs, was not notable. (b) When Suv39h1 was analyzed at a single cell level, the fraction of high-Suv39h1-expressing cells (mainly in the iPSC clusters) was increased slightly in the stretching group (~6% vs ~2% ctrl).

Supplementary Table 1. Information on antibodies

Ab name	Company	Catalog number	Concentration
NMM-HC	bioLegend	909801	1:250
pMLC	Cell signaling	3671s	1:100
vinculin	SANTA CRUZ	sc-73614	1:200
β-catenin	abcam	ab32572	1:100
E-cadherin	SANTA CRUZ	sc-7870	1:200
LaminA/C	abcam	ab108595	1:200
β-tubulin	Cell signaling	2146	1:50
H3K9me3	Cell signaling	13969s	1:1000
Sox2	abcam	ab97959	1:200
Oct4	SANTA CRUZ	sc-5279	1:400
